# Targeted metagenomic recovery of four divergent viruses reveals shared and distinctive characteristics of giant viruses of marine eukaryotes

**DOI:** 10.1098/rstb.2019.0086

**Published:** 2019-10-07

**Authors:** David M. Needham, Camille Poirier, Elisabeth Hehenberger, Valeria Jiménez, Jarred E. Swalwell, Alyson E. Santoro, Alexandra Z. Worden

**Affiliations:** 1 Monterey Bay Aquarium Research Institute, Moss Landing, CA 95039, USA; 2 Ocean EcoSystems Biology Unit, RD3, GEOMAR Helmholtz Centre for Ocean Research, Kiel, 24105, Germany; 3 School of Oceanography, University of Washington, Box 357940, Seattle, WA 98195, USA; 4 Department of Ecology, Evolution and Marine Biology, University of California, Santa Barbara, CA 93106, USA

**Keywords:** virus–host mutualism, *Mimiviridae*, uncultivated giant viruses

## Abstract

Giant viruses have remarkable genomic repertoires—blurring the line with cellular life—and act as top–down controls of eukaryotic plankton. However, to date only six cultured giant virus genomes are available from the pelagic ocean. We used at-sea flow cytometry with staining and sorting designed to target wild predatory eukaryotes, followed by DNA sequencing and assembly, to recover novel giant viruses from the Pacific Ocean. We retrieved four ‘PacV’ partial genomes that range from 421 to 1605 Kb, with 13 contigs on average, including the largest marine viral genomic assembly reported to date. Phylogenetic analyses indicate that three of the new viruses span a clade with deep-branching members of giant *Mimiviridae*, incorporating the *Cafeteria roenbergensis* virus, the uncultivated terrestrial Faunusvirus, one PacV from a choanoflagellate and two PacV with unclear hosts. The fourth virus, oPacV-421, is phylogenetically related to viruses that infect haptophyte algae. About half the predicted proteins in each PacV have no matches in NCBI nr (*e*-value < 10^−5^), totalling 1735 previously unknown proteins; the closest affiliations of the other proteins were evenly distributed across eukaryotes, prokaryotes and viruses of eukaryotes. The PacVs encode many translational proteins and two encode eukaryotic-like proteins from the Rh family of the ammonium transporter superfamily, likely influencing the uptake of nitrogen during infection. cPacV-1605 encodes a microbial viral rhodopsin (VirR) and the biosynthesis pathway for the required chromophore, the second finding of a choanoflagellate-associated virus that encodes these genes. In co-collected metatranscriptomes, 85% of cPacV-1605 genes were expressed, with capsids, heat shock proteins and proteases among the most highly expressed. Based on orthologue presence–absence patterns across the PacVs and other eukaryotic viruses, we posit the observed viral groupings are connected to host lifestyles as heterotrophs or phototrophs.

This article is part of a discussion meeting issue ‘Single cell ecology’.

## 1. Introduction

Viruses have typically been characterized as ‘simple’ pathogens that are entirely dependent on cellular life for production of progeny. However, giant viruses of eukaryotes belonging to the Nucleocytoplasmic Large dsDNA viruses, NCLDV (which include the proposed order ‘Megavirales’ [1]) have led to a re-write of this definition owing to the discovery that they encode multiple functions previously thought to be unique to cellular life [2,3]. These giant viruses are a source of genetic novelty [4] and encode a variety of translational proteins such as translation initiation and elongation factors, tRNA synthetases and tRNAs that had been considered hallmarks of cellular life [3,5,6]. Their genome sizes can exceed those from free-living bacteria and even small pathogenic eukaryotes [7,8], with the often cited minimum genome size cut-off for a giant virus being 300 Kb [9,10]. New information on the diversity of giant viruses and the proteins they encode is providing insight into the evolution of viruses and their influence on host cellular functions [3]. Further, once a greater number of genomes from diverse giant viral lineages are available, it should be possible to advance our understanding of the evolution of cellular life as well, given the proposed importance of viruses in the evolution of eukaryotes and more generally host gene content [11].

In the ocean, viruses are thought to exert significant top–down influence on microbial eukaryotes and have been implicated in the termination of mono-specific phytoplankton blooms [12–14]. Most of the cultivated marine eukaryotic viruses infect phytoplankton, especially prasinophyte or haptophyte algae, and have genomes ranging from 173 to 668 Kb [15–18]. Among these are five giant viruses of marine pelagic phytoplankton [17–23], with complete genome sequences. Additionally, one giant virus of a cultivated heterotrophic marine pelagic protist, the stramenopile *Cafeteria roenbergensis* is available [24]. The only other sequenced pelagic giant virus comes from the uncultured choanoflagellate *Bicosta minor*, as discussed below [25]. Among these viruses, all but one (a *Emiliiania huxley* virus) belongs to a broadly defined family *Mimiviridae* [26,27]. Knowledge about the biology and infection dynamics of smaller marine viruses of eukaryotes is considerable, again, coming from phytoplankton having many cultured representatives available for isolating viruses from the field, such as prasinophytes [18,28–31]. Notably, while the six cultured marine giant viruses reveal extensive novelty, they do not rival the genome sizes of giant NCLDV isolated from other environments such as marine sediments, freshwater systems or soils, which extend to 2.7 Mb in size [3,7].

Cultivation-independent techniques are important in studying eukaryotic viruses in the ocean, owing to the difficulty of cultivating their hosts [25,32,33]. Recently, several giant NCLDV partial genomes were assembled using traditional metagenomic methods from (non-marine) waste waters [5], which have limited diversity, and from deep sea hydrothermal vent sediments with largely uncharacterized microbial communities [34]. Few cultivation-independent studies have been published that capture giant viruses in pelagic aquatic systems. One study, based on bulk metagenomics (i.e. biomass collected by filtering onto a membrane followed by DNA extraction), assembled two related giant virus metagenomes by sequencing Antarctic lake water (Organic Lake) during an algal bloom [35], and argued, owing to similarities to a cultured haptophyte virus, that these viruses infect a haptophyte, also the most abundant algal type present in the sample.

Targeted metagenomics [36], where cells or viral particles themselves are separated by flow cytometry and then sequenced, has led to recovery of partial eukaryotic virus genome assemblies, albeit lacking host information [37,38]. Application to uncultured bacterial cells has provided evidence of co-associations between bacterial hosts and phages during infection [39–41]. Presumably, the viral genomes are effectively ‘pre-amplified’ by the virus having replicated in the sorted host cell, a factor that may improve recovery of viral genomes. Recently, just that has been shown using single-cell eukaryotic metagenomics [25]. This study rendered the discovery of the largest marine NCLDV genome yet reported. The 870 Kb assembly was obtained from a sorted choanoflagellate, a bacterivore (heterotrophic predatory protist) from the Pacific Ocean [25]. The described viruses, ChoanoV1 and ChoanoV2 each encode three microbial rhodopsin proteins, adding to the one previously found in the genome of an algal virus, and two in metagenomic assemblies of putative algal viruses [42]. Unlike the latter, the ChoanoViruses also encode genes for the chromophore, retinal, and therefore may confer phototrophic capacities to their heterotrophic hosts [25].

Here, differential staining, via Lysotracker Green—which stains food vacuoles and/or acidic components of heterotrophic protists [25,43]—and flow cytometry were used to separate individual or multiple living protists from a mixed microbial assemblage in the eastern North Pacific (ENP) Ocean to generate targeted metagenomes [25,36]. We assembled partial viral genome sequences that provide evidence for four deep-branching viruses within the *Mimiviridae*, with total assembly sizes from 421 to 1605 Kb, including the largest viral genome yet recovered from the pelagic ocean. We characterize these assembled genome fragments, explore similarities with the other known NCLDV and analyse their transcriptional patterns from bulk metatranscriptomes to further understand the evolution and ecology of marine giant viruses.

## 2. Methods

### (a) Sampling and flow cytometry

**Figure 1.**
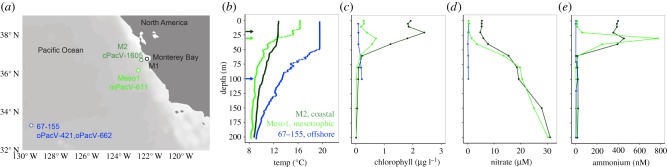
Sites of sorting in distinct ecological zones of the eastern North Pacific Ocean. (*a*) Location of cell sorting experiments, as well as one station where a sample for metatranscriptomics was collected (M1). Depth profiles of (*b*) temperature, (*c*) chlorophyll concentration, (*d*) nitrate concentration and (*e*) ammonium concentration at Stations 67-155 (oligotrophic, blue), Meso1 (mesotrophic, light green) and M2 (coastal, dark green). The arrows indicate the depth from which water was collected for flow cytometric sorting, specifically, the deep chlorophyll maximum for 67-155 (100 m, see electronic supplementary material, figure S1a for *in vivo* fluorescence profile) and at the sub-surface maxima for Meso1 (30 m) and M2 (20 m). Note, 67-155 data in (*b*), (*c*) and (*d*) are from a CTD cast executed 8 h prior to the cast on which sorting was performed. Additionally, ammonium concentrations at 67-155 (*e*) were measured from a CTD cast collected 25.5 h prior to sorting. Electronic supplementary material, figure S1e,f provides additional data on temperature and salinity for the cast on which cell sorting was performed, which exhibited highly similar conditions to casts depicted in the above panels.

Samples were collected from the depth where the chlorophyll maximum was observed at three sites in the ENP from a coastal (M2, 20 m depth), mesotrophic (Meso1, 30 m depth) and oligotrophic (67–155, 100 m depth) station (figure 1*a*; electronic supplementary material, table S1). Seawater was collected using Niskin bottles mounted on a rosette package including a conductivity, temperature, and depth instrument (CTD) and fluorometer. Chlorophyll concentrations were quantified via filtration onto a GF/F filter and acetone extraction [44] from eight discrete depths including those of the cell sorts as described below. Nitrate, phosphate and silicate concentrations were determined colorometrically [45] and ammonium was determined fluorometrically [46].

For flow cytometric sorting, whole seawater was pre-filtered with a 30 or 40 µm nylon mesh and concentrated by gravity over a 0.8 µm pore size filter (10-, 50- or 100-fold depending on the sample; electronic supplementary material, table S1). Concentrated samples were stained with Lysotracker Green DND-26 (Life Technologies Catalogue #L7526), a fluorescent probe that accumulates in acidic compartments [43], with 25 or 100 nM final concentration for 15 min in the dark (electronic supplementary material, table S1) [25]. Samples were then run on an Influx Fluorescence Activated Cell Sorter (BD Biosciences) equipped with a 488 nm laser, running sterile 1× PBS as sheath fluid. Eukaryotic cells possessing Lysotracker signal, detected as green fluorescence collected through a 520/35 nm (67-155 and M2) or 531/40 nm (Meso1) bandpass filter, were sorted into tubes or wells (300 cells per tube for 67-155 and Meso1, or individual cells into wells of a 384-well plate at M2). A NOT logic gate encompassing the natural chlorophyll signal of phytoplankton was used to exclude photosynthetic cells in order to more specifically target heterotrophic protists. Sort tubes and plates were frozen at −80°C immediately after cell sorting.

We focused on sorts in which partial NCLDV genomes were obtained, two from single cell sorts (Station M2, 20 March 2014) and two from multi-cell sorts (Station 67-155, 11 October 2013; Station Meso1, 16 September 2012).

### (b) Assembly and genome identification

Multi-cell sorts and single cells were amplified via multiple displacement amplification (MDA) [47] and libraries were sequenced with paired-end Illumina HiSeq (electronic supplementary material, table S1). Paired reads were quality filtered with Trimmomatic v. 0.36 [48] by truncation at the first base below a *q*-score of 3, and then by truncation below a quality score of 30 across a moving average of 25 bp (full settings: LEADING:3 TRAILING:3 SLIDINGWINDOW:25:30 MINLEN:50). Quality-filtered reads, including paired and unpaired reads, were then assembled with Spades v. 3.11.1 with -k 21,33,55,77,99,127 via the single-cell option (‘-sc’) [49]. Contigs longer than 1 Kb were then selected for downstream processing. Notably, however, contigs less than 5 Kb made up only a small fraction of the partial genome assemblies (see §§3,4 and electronic supplementary material, Discussion). Quality-filtered paired reads from each sample were mapped back to the contigs with Bowtie 2, using default settings [50]. Binning of contigs was performed in Anvi'o [51] based on similarities in tetranucleotide frequency and GC-content. Protein coding sequences were predicted via Prodigal [52] from each contig and then searched for any of 47 putatively ancestral NCLDV genes [53] via hmmscan (*e*-value < 10^−50^) [54] (electronic supplementary material, table S2). To generate the hidden Markov models (hmm) of the 47 proteins, the proteins were collected from the nucleocytoplasmic viral orthogroup (NCVOG) dataset as previously reported [53]. Then, for each NCVOG, the sequences were aligned with MAFFT (–auto) [55]. Hmms were then constructed with hmmbuild and prepared for searching with hmmpress. The alignments, hmm models and the full package for use in Anvi'o are available via FigShare (doi:10.6084/m9.figshare.9108335). A visualization of contigs that contained any of the putatively ancestral NCLDV genes was added to the Anvi'o interactive interface to aid in identification of NCLDV contigs. These same hmm models were also used to assess the number of these proteins in the other representative NCLDV as reported in electronic supplementary material, table S2 via hmmscan with an *e*-value cut-off of less than 10^−50^. Additionally, each contig was searched for rRNA gene sequences via barrnap [56], which were added to Anvi'o visualizations to identify obviously cellular contigs. After genome binning, the largest viral genome recovered (cPacV-1605, see §3) was secondarily assembled in Geneious v. 6.1.6 [57]; contigs that overlapped by more than 100 bp with 100% identity were assembled, accordingly. This secondary assembly resulted in a reduction in the number of contigs from 29 to 14. The other viruses (for which the initial assemblies had fewer contigs) were not further assembled in this manner.

To determine the identity of the sorted potential host cells from each of the metagenomic samples, we used metaxa2 [58] to extract paired reads that were of 16S or 18S rRNA gene sequence origin. In the ‘paired-end’ format, metaxa2 identifies read pairs if either of the pairs are rRNA gene sequences. Therefore, to improve the classifications of the extracted rRNA gene sequences, we only further classified pairs from which both reads were derived from rRNA gene sequences. To do this, a secondary filter was performed on each read by individually searching the SILVA 132 database [59] with blastn (*e*-value < 10^−25^). Then, the paired reads for which both were of rRNA gene sequence origin were combined with an N between the two ends and classified via the *assign_taxonomy.py* command in QIIME [60] with the RDP classifier [61] using the SILVA 132 database as a reference. The kmer-based ribosomal database project (RDP) classifier ignores the degenerate base (‘N’) [62] making it suitable for this analysis. Krona visualizations of the rRNA classification results were generated with the *ktImportText* command of the krona package [63].

For the two single cells sorted from Station M2 we performed additional searches to gather information about the identity of the sorted cells. For one of the two single-cells, no rRNA genes were detected (a sort from Station M2). Therefore, each assembled contig (greater than 1 Kb) was classified with the CAT annotation tool [64]. We also performed an additional blastx search of the contigs against NCBI nr and a dataset of 19 choanoflagellate transcriptomes [65] (sequence match across 30% amino acid similarity over more than 100 aa, and bit-score greater than 100). Each cell was also searched by blastn against a draft genome of *B. minor* (choanoflagellate), which is assembled in [25].

### (c) Virus genome annotation

Annotation of predicted proteins was performed via hmmscan searches of the Pfam database [66] (*e*-value < 10^−5^). To identify orthologous NCLDV proteins, we evaluated 81 partial and complete representative NCLDV genomes (electronic supplementary material, table S3), including the PacVs, with Orthofinder v. 1.1.8 [67]. In order to infer functional characteristics of the various lineages across NCLDV, the orthofinder results were imported into R [68] and the viruses were clustered based on the patterns of presence and absence of orthogroups with pvclust [69] and 500 bootstraps. We used the approximately unbiased *p*-values for bootstrap support. Clustering patterns and distributions of orthogroups were visualized via the superheat R package [70]. Circular genome maps were produced in R via the circlize package [71] (the order of contigs is arbitrary). Moving averages of GC-content, for genome maps, were calculated in R with the Biostrings package [72]. Rhodopsin functional motifs and predicted spectral tuning were determined based on relevant positions as previously described [73–76].

### (d) Phylogenomics and phylogenetics

A phylogenomic reconstruction based on ten putatively vertically transmitted core NCLDV genes was generated as previously described [25]. Five of these proteins overlap with those used previously for NCLDV phylogenomic analyses [5], specifically, DNA polymerase elongation subunit family, D5-like helicase-primase, packaging ATPase, Poxvirus Late Transcription Factor VLTF3-like, and DNA or RNA helicases of superfamily II. The other five were selected as part of analyses in [25], specifically, RNA polymerase, subunit alpha, RNA polymerase subunit beta, mRNA capping enzyme DNA topoisomerase II and YqaJ viral recombinase. All 10 orthologues were found for one of the new viruses (oPacV-421), nine in cPacV-1605 and mPacV-611, and seven in oPacV-662. Additionally, we identified five or more of these orthologues in the recently reported NCLDV from deep sea sediments LCMiAC01, LCMiAC02, LCMAC102, LCMAC103, LCPAC104, LCPAC201 and LCPAC202 [34] and the soil viruses Harvfovirus, Satyrvirus, Terrestrivirus, Hyperionvirus, Edafosvirus and Faunusvirus [38]. All new sequences were added to the single-gene alignments, re-aligned, manually inspected and trimmed of ambiguously aligned positions as previously described [25], resulting in a 67-taxa matrix of 4424 amino acid (aa) residues. A maximum-likelihood tree was inferred by IQ-TREE v. 1.5.5 [77] using the C20 empirical mixture model in combination with the LG matrix, amino acid frequencies computed from the data and four gamma categories for handling the rate heterogeneity across sites (LG+C20+F+G model). The best tree under this model was used as a guide tree to estimate the ‘posterior mean site frequencies’ [78]. This LG+C20+F+G-PMSF model was finally used to re-estimate a maximum-likelihood tree and for a nonparametric bootstrap analysis with 500 replicates.

A phylogenetic reconstruction was also performed using only the Family B DNA Polymerase protein (PolB) (electronic supplementary material, table S3). For the single gene phylogeny, PolB alignments were made via MUSCLE [79] and positions with greater than 20% gaps were removed via trimAl (-gt 0.8) [80], resulting in a final alignment of 869 aa positions. Phylogenetic analysis was performed with IQ-TREE using 1000 ultrafast bootstraps [81] with the evolutionary best model selected via standard model selection (TEST option) [82] resulting in the best-fit model (LG+F+I+G4).

For the phylogenetic reconstruction of Amt/MEP/Rh superfamily proteins, 19 907 unaligned protein sequences were downloaded from [83] (https://zenodo.org/record/61901#.XRui-ZNKjUI). These sequences consisted of 15 378 dereplicated Amt/MEP/Rh sequences from UniProt100 [84], 4446 sequences from the Marine Microbial Eukaryote Sequencing Project (MMETSP) [33] and 83 sequences from protist genome sequencing projects at the DoE-Joint Genome Institute. In addition to these sequences, metatranscriptomes from 19 choanoflagellate species [65] were searched via hmmscan for the ammonium transporter Pfam domain (PF00909), resulting in addition of 100 Amt/MEP/Rh sequences to the database. Finally, the ammonium transporter sequence from the *Ostreococcus tauri* virus 6 (OtV6), and the two PacV protein sequences with protein domains matching the Amt/MEP/Rh superfamily proteins were added to the dataset. The total dataset included 20 010 sequences. The sequences were aligned with MAFFT [55] using default settings. The alignment was then filtered with trimal, removing positions that contained more than 50% gaps (-gt 0.5) [80]. Then, poorly aligned and/or false positive sequences were removed with trimal with settings of –resoverlap 0.6 and –seqoverlap 60, resulting in 17 339 sequences and 374 positions. An Amt/MEP/Rh phylogeny was then built with FastTree [85], using default settings, and imported into the iTol server for visualization [86]. Relevant groupings (Amt-Euk, Mep, Rh, Rh-a, Rh-b, Rh-c) were based on those described previously [83,87,88]. From this phylogeny, the clade containing the Rh sequences were extracted at a node with 91% bootstrap support (as indicated in §3). The resultant 1532 sequences were aligned with MAFFT, filtered with trimal and the phylogeny constructed as before, except with the -slow setting of FastTree. The total number of amino acid positions in the Rhesus family alignment was 362. Sequence alignments and tree files for the trees shown in the paper are available via Figshare (doi:10.6084/m9.figshare.9722807).

### (e) Mapping of reads from metatranscriptomes

Metatranscriptomic and metagenomic reads for the multi-cell and single cell sorts from the ENP (electronic supplementary material, table S1) were quality filtered as described above and then mapped with BBMap.sh [89], using default settings (ambiguous reads mapped to the first best site) and with a similarity requirement of 99%. Read counts for each predicted protein were summarized with HTSeq [90]. Tara Oceans metatranscriptomic reads [91] from 84 samples (electronic supplementary material, table S4) from the protistan size fraction were quality filtered (as described above) and searched against the predicted proteins from the viral references (indicated in electronic supplementary material, table S3) with DIAMOND blastx [92]. Sequence reads with a bit-score greater than 50 to any of the viruses were subsequently searched against NCBI nr by DIAMOND blastx. These results were compared to the initial matches and, again, only the single best match for each metatranscriptomic read was retained. In cases where a query read had multiple best hits (ties) to a reference sequence, the reported reference match was chosen randomly. The results from all stations were combined and then the read counts for each gene plotted in R [68] with ggplot [93].

## 3. Results

### (a) Sampling of distinct oceanographic zones

The seawater samples collected came from an oligotrophic region at the edge of the North Pacific Subtropical Gyre (Station 67-155), a region south of the Monterey Bay (Meso1) and a site at the mouth of the Monterey Bay (Station M2) (figure 1*a*). We observed a deep chlorophyll maximum at 100 m and low nutrient concentration at 67-155 (figure 1*a*–*e*; electronic supplementary material, figure S1). The second station (Meso1, figure 1) had intermediate nutrient concentrations and phytoplankton biomass (inferred from chlorophyll concentrations) compared to the other two sites. Station M2 had the highest measured chlorophyll at the time of sampling (2.5 µg l^−1^). The values and characteristics we observed fell within those of prior studies on this oceanographic region (e.g. [94–96]).

### (b) Taxonomic characterization of sorted cells

Cells were concentrated, stained with the acidic vacuole stain Lysotracker Green (to exclude free-living bacteria) and flow cytometrically sorted with selection including a gate to exclude photosynthetic cells based on their chlorophyll fluorescence (electronic supplementary material, figure S2a–c) [25]. To identify the eukaryotes in the Station M2 single-cell sorts from which viruses were recovered (see below) we first searched for rRNA gene sequences. Assemblies from one of the single cell sorts had a full-length 18S rRNA gene sequence with 99% similarity to *B. minor*, an uncultivated choanoflagellate, while the other had no rRNA gene sequence. The recovery of the *B. minor* rRNA gene sequence was consistent with the observation that 95% of cells sorted on this date and station were *B. minor* [25]. From the cell with the *B. minor* 18S rRNA gene sequence, 11% of quality filtered reads mapped to a *B. minor* draft genome [25] at high stringency. Furthermore, 686 of 1196 total contig assemblies were more than 95% similar to a draft *B. minor* genome; of these, 238 had best blastx hits to NCBI nr, supplemented with a dataset of choanoflagellate transcriptomes [65] (and excluding *B. minor*), to other choanoflagellates or other opisthokonts (electronic supplementary material, figure S3a). For the cell without an 18S rRNA gene sequence in the metagenomic assemblies, only 1.1% of reads mapped to the *B. minor* draft genome, and only four of 452 contigs matched. Furthermore, none of the remaining contigs after excluding *B. minor* associated contigs, had best hits to choanoflagellates or opisthokonts. Rather, most contigs had best hits to bacteria (Flavobacteria) and phage (electronic supplementary material figure S3b). Hence, one of the sorted single cells with cPacV-1605 was *B. minor*, while the identity of the other sorted eukaryote that contained this virus could not be verified.

The multi-cell sorts also targeted heterotrophic protists, but not a single population. To characterize the suite of potential associations, we analysed the rRNA gene sequences from the unassembled multi-cell sorts. The multi-cell sort at 67-155 appeared to contain mostly rRNA gene sequences from alveolates, especially the Syndiniales, most of which have as yet unknown trophic roles [97,98]. At this station 92% of all rRNA genes sequences (i.e. both 16S and 18S rRNA genes) in the multi-cell sort were from Syndiniales I or II (electronic supplementary material, figure S4a). Smaller contributions were observed for other alveolates, such as Peridiniales, an order of dinoflagellates containing heterotrophs and autotrophs (3.5%) (electronic supplementary material, figure S4a). Rhizaria were also detected, specifically Retaria (1%). The remaining approximately 1% was composed of other eukaryotes, including choanoflagellates, and bacteria (electronic supplementary material, figure S4b). In contrast, the Meso1 multi-cell sort was dominated by stramenopiles, especially MAST-4 (52%), which are known to be present at all the sites studied herein [99]. Although MAST-4 are uncultured they have been shown to be heterotrophic predators that actively phagocytose other microbes [100,101]. The Meso1 multi-cell sort also included alveolates (Syndiniales, 29%) and opisthokonts (Capsasporidae, 15%, a phylogenetic group sister to choanoflagellates and animals [102,103]), with a minor contribution by Proteobacteria (0.8%) (electronic supplementary material, figure S4c,d).

### (c) Giant viruses in cell sorts

Giant viruses were identified in each of the described single- and multi-cell sorts through clustering of assembled contigs based on tetranucleotide frequency as well as identifying matches to 47 putatively ancestral proteins of the NCLDV group, and verifying absence of rRNA gene sequences [53] (electronic supplementary material, figure S3 and S5–S7). One virus was recovered from the coastal environment (M2, where we observed the same virus twice; see below), one from the mesotrophic site (Meso1), and two from the edge of the North Pacific gyre at Station 67-155. The identified viruses ranged in size from 421 to 1605 Kb (table 1). The viruses are hereafter referred to as cPacV-1605, mPacV-611, oPacV-421 and oPacV-662, where the letters ‘c' (coastal, M2), ‘m' (mesotrophic, Meso1) and ‘o’ (oligotrophic, 67-155) indicate the environment from which they were recovered, PacV refers to the biome in which they were identified (Pacific Ocean) and the number refers to the length of the recovered partial genome sequence (in Kb).

**Table 1.** Genome statistics of the four partial genome assemblies from newly discovered pelagic marine giant viruses.

virus	longitude, latitude	genome assembly (bp)	contig number	largest contig	predicted proteins	eukaryote sorted^a^
cPacV-1605	36.688 N, 122.386 W	1 605 493	14	363 384	1549	*Bicosta minor*
mPacV-611	36.144 N, 122.570 W	610 889	7	228 203	574	300 cells^b^
oPacV-662	33.292 N, 129.419 W	662 110	18	150 190	635	300 cells^c^
oPacV-421	33.292 N, 129.419 W	420 509	13	124 072	429	300 cells^c^

^a^Sorted eukaryotes and estimated relative abundances from multi-cells sorts identified via classification of 18S and 16S rRNA gene sequence reads.

^b^MAST-4 (52%), Syndiniales (29%), Capsasporidae (15%), Proteobacteria (0.8%).

^c^Syndiniales (92%), Peridiniales (3.5%), Rhizaria (1%), less than 1% Choanoflagellates, other eukaryotes and bacteria.

We assessed the assembly quality for all four PacV partial genomes by calculating N50 [104], which was between 124 and 363 Kb (table 1). All four PacVs assemblies had fewer than 18 contigs (mean contig number = 13, table 1) and the mean size of these contigs was 63 442 bp. Only oPacV-662 had a notable number of smaller contigs, between 1 and 2 Kb, which made up 0.4% of the genome, while oPacV-662 and oPacV-421 had just 2 and 3 contigs between 2 and 5 Kb making up 1.3% and 3% of their partial genome sequences, respectively. For cPacV-1605, two highly similar partial genomes were recovered from the single cell sorts at Station M2. One assembly was 1.2 Mb (33× coverage), coming from the sorted *B. minor* cell (electronic supplementary material, figure S3a), and the other was 1.6 Mb (621× coverage), coming from the well with few identifiable eukaryotic contigs (electronic supplementary material, figure S3b). These were termed cPacV-1605, for the larger genome assembly, because the two viruses had an average nucleotide identity of 99.3% and the larger assembly was in fewer contigs (11 versus 119) (electronic supplementary material, figure S3a). Additionally, 91.2% of the larger PacV-1605 was covered by reads in the other well at 40.5× coverage (electronic supplementary material, figure S8). Hereafter, we consider only the larger genome assembly, unless specifically stated otherwise, and we conclude the cPacV-1605 host is *B. minor*.

All four final PacV assemblies had distinct tetranucleotide frequencies and lower GC-content (25–31%) compared to co-sorted cells (electronic supplementary material, figures S3 and S5–S7; table 1). In addition, the coverage of each of the viral genome sequences recovered from the different sorts was high (table 1), specifically 621×, 197×, 64× and 228× for cPacV-1605, oPacV-662, mPacV-611 and oPacV-421, respectively (table 1). The amount of single nucleotide variation for each was less than 1 bp per Kb (electronic supplementary material, figures S3 and S5–S7).

### (d) Phylogenetic relationships and gene content

The number of the previously recognized 47 putatively ancestral-NCLDV-proteins found in the PacV genome assemblies was between 28 and 35 (electronic supplementary material, figures S3 and S5–S7; table 1; electronic supplementary material, table S2). These numbers are in line with those from previously sequenced *Mimiviridae* genomes, all of which have fewer than 47 of these putatively ancestral proteins (electronic supplementary material, table S2) [53]. For cultured *Mimiviridae* with complete genome sequences 31 are present on average (range 14–39, electronic supplementary material, table S2).

**Figure 2.**
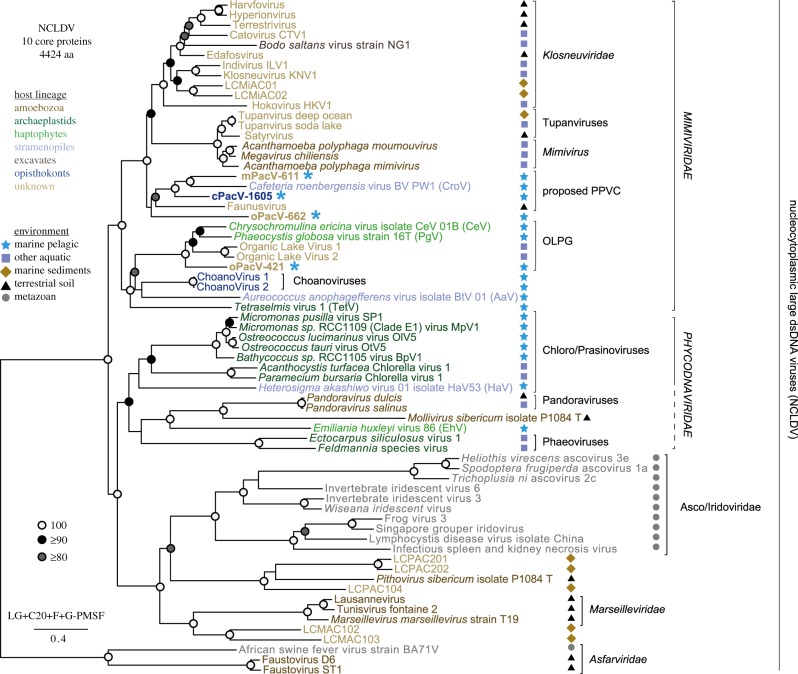
Maximum-likelihood phylogenomic reconstruction of NCLDV based on 10 putatively vertically inherited proteins. Support is indicated when greater than 80% (500 bootstrap replicates). The scale bar and the number beneath it indicate the estimated number of substitutions per site and the model for tree reconstruction is provided. Abbreviated names used in the manuscript for cultured marine pelagic viruses are shown in parentheses: *Cafeteria roenbergensis* virus BV PW1 (CroV), *Heterosigma akashiwo virus* 01 isolate HaV53 (HaV), *Phaeocystis globosa* virus strain 16T (PgV), *Chrysochromulina ericina* virus isolate CeV 01B (CeV), *Aureococcus anophagefferens* virus isolate BtV 01 (AaV) and *Emiliania huxleyi* virus 86 (EhV). Note that viral naming conventions are currently an active area of research and discussion, for example one recent proposed taxonomy suggested that the shared ancestry and common traits of the nucleocytoplasmic large DNA virus (NCLDV) group justified renaming these viruses as a formal order called ‘Megavirales’ [1]. Additionally, there was a proposal that the *Mimiviridae* groups be reclassified into proposed subfamilies, Megamimivirinae and Mesomimivirinae (the latter mostly being giant algal viruses and CroV) [105]. Note that the proposed Mesomimivirinae was split by our 10-gene phylogeny. Because these groups have not yet been approved by the International Committee on the Taxonomy of Viruses (ICTV) they have not been used herein. We simply refer to the previously unrecognized clade that brings together CroV, Faunusvirus, mPacV-611 and cPacV-1605, as being the predatory protist viral clade (PPVC), until viral classification conventions can be resolved. OLPG, Organic Lake Phycodnaviridae group.

In an effort to understand the phylogenetic relationships among the viruses we analysed 10 of the 47-protein ancestral set, all of which are thought to be vertically inherited. The tree contained a moderately supported broad clade with relatively deeply branching members (figure 2) within the broad family *Mimiviridae* [26]. This clade is clearly distinct from other major named giant virus groups within the *Mimiviridae*, such as Mimiviruses, Tupanviruses, Klosneuviruses, ChoanoViruses and Organic Lake Phycodnaviridae Group. This newly delineated clade incorporates cPacV-1605, mPacV-611, the previously described predatory stramenopile-infecting virus CroV and the metagenomic-assembled Faunusvirus. PacV-662 has a less well-resolved placement lacking bootstrap support (figure 2; electronic supplementary material, figure S9), but appears to affiliate with the clade containing cPacV-1605 and mPacV-611. To avoid confusion in the future, as viral taxonomy and nomenclature is an active area of research, we simply refer to this likely family-level clade as the PPVC (predatory protist viral clade) for further discussion herein. The fourth virus, oPacV-421, formed a highly supported clade with the *Phaeocystis globosa* virus (PgV), and *Chrysomchromulina ericina* virus (CeV), and two putative haptophyte viruses from metagenomic data. Branch lengths indicated cPacV-421 was relatively closely related to these viruses, at least based on our 10-protein analysis.

We also analysed the Family B DNA Polymerase, the only protein found in a single copy in all available gapless genome assemblies [53,105,106] and one for which there are many additional viral sequences available from the environments because they have been captured in PCR-based clone library studies targeting viral PolB [106]. The topology of this tree was similar to the multi-protein tree (figure 2; electronic supplementary material, figure S9).

**Figure 3.**
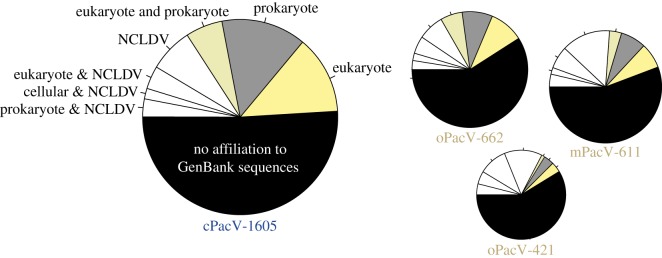
Summarized taxonomic affiliation of predicted proteins in the four novel giant NCLDV. Taxonomic affiliations to prokaryotes (combining archaea and bacteria), eukaryotes or NCLDV were determined based on the top ten DIAMOND blastp hits (*e*-value < e^−5^). If among these top ten there were hits to more than one lineage, it was categorized accordingly. Pie chart area is proportional to recovered genome size.

At the genome level, the PacVs were quite unique with over half of their proteins having no sequence match in NCBI nr (ranging from 252 to 790, in oPacV-421 and cPacV-1605, respectively) (figure 3). This totalled to 1735 new hypothetical proteins across the four PacVs. Among the proteins with similarities in databases, we found that the matches were roughly equally distributed between highest affiliation (based on blastp) to eukaryotes, bacteria, and NCLDV viruses (figure 3).

**Figure 4.**
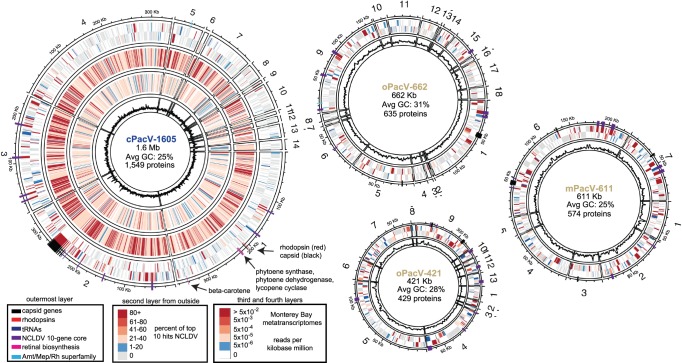
Genomic visualization of the novel giant NCLDV viruses. The outermost coloured layer (intersecting with contig map) shows the location of the notable genes indicated in the legend. The second layer shows the predicted proteins of each virus according to the coding strand; the colour represents the percent of the top 10 NCBI nr matches that were best hits to other NCLDV viruses. For virus cPacV-1605, the third and fourth layers show the reads per Kb million of each predicted gene that were recovered from metatranscriptomes collected at the time of sampling and one month prior at an ocean location approximately 30 km away (M1, figure 1*a*). The innermost layer in each figure shows a 1 Kb moving average of GC-content, where the scale is set from 0% to 60% GC-content. Contigs that are less than 5 Kb in length (three and four for oPacV-421 and oPacV-662) are marked with a dash.

The four PacVs encoded some of the ‘hallmark’ translational genes and genes encoding other cellular machinery shared with cellular life. The total number of predicted proteins ranged from 429 to 1549 and scaled with the PacV partial genome sizes (figure 4). The two smaller PacVs each have six tRNAs, oPacV-662 has 22 and cPacV-1605 has 51 (figure 4), the latter of which is the second most recovered from a giant virus, topped only by the Tupanvirus from deep ocean sediments [6]. All four viruses have genes encoding nucleotide and amino acid synthesis-related proteins, including thymidylate synthase. Asparagine synthase, responsible for converting aspartate to asparagine and common in *Mimiviridae* and prasinoviruses [109], was present in all PacVs except oPacV-421. Each of the viruses also encoded translation initiation factors, from one in oPacV-421 to eight in cPacV-1605, and all but oPacV-421 encoded elongation factors, with up to 12 in cPacV-1605. Three of the four viruses encoded tRNA synthetases, with oPacV-421 and mPacV-611 encoding one each, and cPacV-1605 encoding 15.

**Figure 5.**
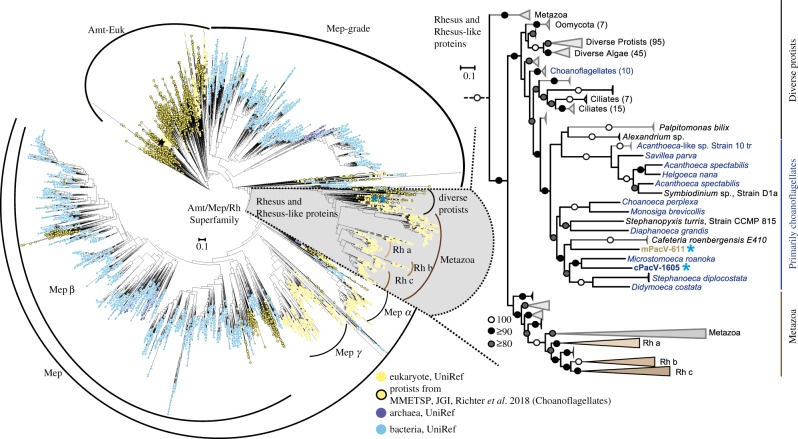
Phylogenetic analysis of the Amt/MEP/Rh superfamily. (*a*) Phylogenetic reconstruction of 17 339 proteins predicted to have a domain matching PF00909, Amt/Mep/superfamily. As described in §2, the taxon selection is the same as that from [83], supplemented with proteins from 19 choanoflagellates [65]. The total number of amino acid positions analysed was 374. (*b*) Amt/MEP/Rh superfamily reconstruction restricted to the Rh protein family, which includes mostly proteins from animals and diverse protists. The phylogeny includes 1,532 sequences and 362 positions.

Two of the PacVs, cPacV-1605 and oPacV-662, had a protein domain of particular relevance to nutrient cycling in the ocean: eukaryotic-like ammonium transporters (Pfam: PF00909, *e*-value < 10^−60^). These proteins were specifically affiliated with the ammonium transporter/methylammonium permease/Rhesus factor superfamily (Amt/MEP/Rh superfamily), which are cell membrane-bound proteins that transport ammonium and/or ammonia in all three domains of life [110,111]. Here, our large-scale phylogenetic reconstruction of over 17 000 proteins, after early identification and analyses of these proteins in protists [87], and subsequent larger scale analyses [83,88], placed cPacV-1605 and oPacV-662 in the Rh-like family (figure 5*a*) with 91% local support based on the Shimodaira–Hasegawa test. The Rh-specific phylogeny indicated that the cPacV-1605 and oPacV-662 proteins belong to a clade that harboured diverse protists, including choanoflagellates (figure 5*b*) [65] with 97% support. The PacV proteins had approximately 30% amino acid identity to vAmt, an ammonium transporter of a phytoplankton *O. tauri* virus, compared with 36–40% to the well-studied Rh proteins in humans, and 39–43% to the closest related putative Rh sequence from a cultured organism, the choanoflagellate, *Microstomoeca roanoka*, indicating along with the phylogeny, a distinct evolutionary history between the PacV Rh-like proteins and vAmt.

A putative microbial rhodopsin protein is present in cPacV-1605 alongside the proteins required for beta-carotene biosynthesis (phytoene synthase, phytoene dehydrogenase, lycopene cyclase) and the cleavage enzyme that converts beta-carotene to retinal (beta-carotene dehydrogenase) (figure 4). The cPacV-1605 rhodopsin has an as-yet uncharacterized motif type [25,75], DTV, hence its biochemical function is not known, though, like other known VirR, it appears tuned to green light with a methionine in spectral tuning site 105. cPacV-1605 is only the second virus discovered after the ChoanoViruses [25] that encodes the proteins involved in beta-carotene biosynthesis and retinal production.

**Figure 6.**
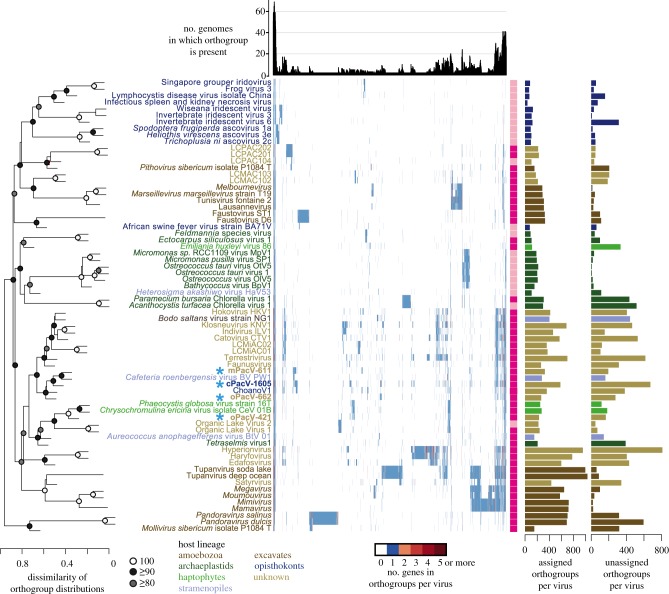
Orthogroup patterns for proteins of PacVs and a broad array of representative NCLDV having sequenced genomes. Hierarchical clustering is based on the presence or absence of all orthogroups by pvclust with 500 bootstraps from the ‘approximately unbiased’ method [69]. The histogram above the heatmap shows the number of genomes in which an orthogroup was found. The light pink and dark pink bars indicate whether or not a particular virus is categorized as a ‘giant’ virus (i.e. genome size greater than 300 Kb). The bar charts at the right indicate the sum of orthogroup proteins found for a given virus and the number of proteins that were not shared with any of the viruses analysed.

In addition to these components shared with cellular life, hundreds of proteins from each of the viruses are homologous to those in other giant viruses. Clustering based on an all-versus-all orthologue presence and absence pattern analysis of a representative set of NCLDV and the four new PacV revealed groupings that deviated from clade structure based on phylogenetic relatedness (figures 2 and 6). Three of the PacVs (oPacV-662, cPacV-1605, mPacV-611) cluster with CroV, *Bodo saltans* virus and other viruses of heterotrophic eukaryotes (known or presumed based on the environmental source, more details below), while oPacV-421 clusters with PgV and other algal viruses (either known or presumed), all with significant bootstrap support (figure 6). Thus, notable differences are apparent between the orthologue presence–absence clusters (figure 6) and the phylogenetic reconstructions (figure 2), with the former bringing together: (1) prasinoviruses and chloroviruses, (2) viruses of other eukaryotic algae, mostly haptophytes (a cluster that includes oPacV-421) and a broad group that includes (3) Mimiviruses, (4) Klosneuviruses and (5) a cluster containing only marine viruses from predatory protists, specifically cPacV-1605, oPacV-662, mPacV-611, CroV and Choanoviruses. Additionally, the latter two orthologue groupings are adjacent and have bootstrap support, so that there is an overarching group incorporating Mimiviruses, Klosneuviruses, CroV and the PacVs. This clustering based on orthologue presence–absence patterns highlights similarities in the metabolic potential of diverse viruses of heterotrophic protists.

### (e) Read recruitment from ocean metatranscriptomes

The high number of newly identified genes in the PacVs raises the question as to whether they are actively transcribed and what their patterns of expression are in the environment. A metatranscriptome generated from the same water sample as cPacV-1605 indicated that at least 85% of the predicted 1549 cPacV-1605 genes were transcribed at the time of sampling. The mapping of these genes, at 99% nucleotide similarity, was generally uniform across individual genes, suggesting that the mapping is specific and not an artefact of highly conserved regions (electronic supplementary material, figure S10a). Likewise, mapping of a traditional ‘bulk’ metagenome to cPacV-1605, from the same station and date, was highly even, further suggesting mapping is specific (electronic supplementary material, figure S10b). Among the genes most highly expressed were capsids, heat shock proteins (HSP), HSP70 and HSP90, and peptidases (electronic supplementary material, table S5). In a metatranscriptome collected from nearby (approx. 30 km away) as well as from one month later (30 km away), the transcriptional pattern was similar (adjusted *R*^2^ = 0.55, *p* = 2.2 × 10^−16^), suggesting the transcriptional pattern during infection is consistent across these two sites (figure 4; electronic supplementary material, table S5).

**Figure 7.**
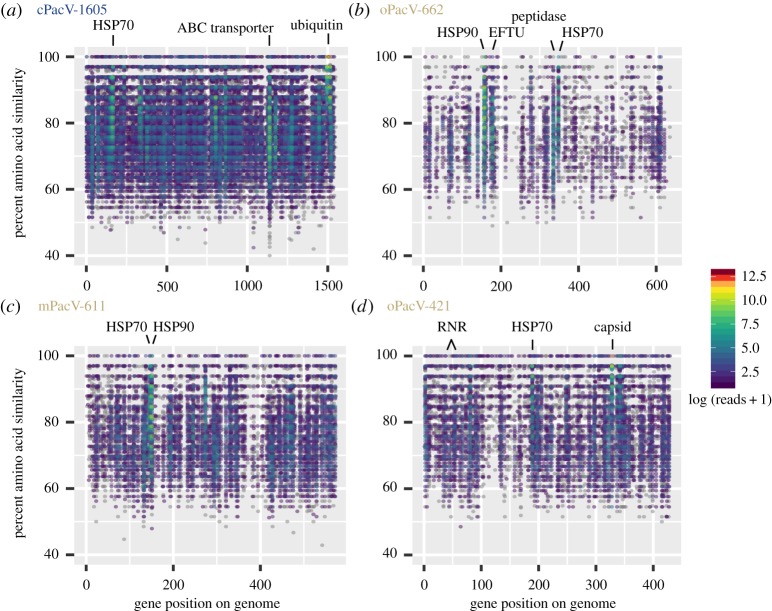
Detection of viral gene expression in metatranscriptomes from other regions. (*a*–*d*) Read recruitment by blastx of Tara Oceans metatranscriptome reads to PacV predicted proteins. Only reads that were a best-hit (and bit-score greater than 50) to a given viral genome compared with a representative set of NCLDV (electronic supplementary material, table S3 and figure S11) and all of NCBI nr are shown. Reads are plotted on a gene-by-gene basis, and reads to each gene were summed across 84 Tara metatranscriptomes. Gene order is the same as shown in figure 4. Abbreviations: EFTU, elongation factor thermo unstable; RNR, ribonucleotide reductase; HSP, heat shock protein.

In addition to this mapping, we explored the distribution and expression patterns of the viruses in the global ocean by querying metatranscriptomic reads from the protistan size fraction of the Tara Oceans dataset [91]. Among a selected set of 38 *Mimiviridae* and *Phycodnaviridae* genomes that were searched, all PacVs ranked within the top eight viruses with respect to recruitment level, as were *Bathycoccus* virus 6 (BpV6), ChoanoV1, CeV and Organic Lake virus 2 (electronic supplementary material, figure S11). The PacVs recruited reads broadly across their partial genomes, with 20% amino acid divergence on average to the recruited Tara metatranscriptome reads (figure 7). In addition to this broad recruitment, a select number of proteins across the partial genomes recruited many more reads. The annotation of these highly expressed proteins varied, with HSP70 and HSP90 being highly expressed across all of the viruses. Additionally, an ABC transporter (no obvious sequenced-based specificity) (cPacV-1605), a peptidase and an elongation factor (oPacV-662), capsid proteins and ribonucleotide reductases (oPacV-421) were among those genes with high relative gene expression (figure 7). Despite the large number of distant matches (60–90% amino acid similarity), there was little mapping at the high sequence similarity (e.g. greater than 90% amino acid similarity), suggesting that the sequenced viruses may have populations constrained by the local habitat (or host distributions), effectively being endemic to the region in which they were recovered.

## 4. Discussion

Environmental sequencing studies suggest that marine giant viruses belonging to the NCLDV may be more diverse in terms of operationally defined taxonomic clusters than bacteria and archaea [112]. However, genome-scale characterizations of this group have been hampered by dependency on cultivation-based approaches for viral isolation, as well as the limitations of traditional metagenomic approaches. Here, we describe the recovery of partial genome assemblies that provide evidence for four novel *Mimiviridae*. The viruses were recovered using cultivation-independent targeted metagenomics [25,36] at ENP sites with very different water column structures and ecology (figure 1*a*, [94,96,99]). The recovery of these new giant virus partial genome assemblies adds significantly to the number of previously genome-sequenced pelagic marine giant *Mimiviridae*.

The PacVs include a virus with the largest partial genome found in the marine environment at 1605 Kb, which was discovered in association with a choanoflagellate. Remarkably, all PacVs have quite low %GC content (25–31%), like CroV (23%) and the ChoanoViruses (22%), making them potentially subject to being missed by a new MDA enzyme [41] that captures high-GC templates efficiently. About 50% of each of the predicted proteins of the PacVs have no known sequence similarity to any other virus or cellular life, reiterating that these viruses are vast genetic reservoirs and that the diversity of *Mimiviridae* is a relatively underexplored frontier of biological diversity.

### (a) Interpretation of viral genomes from targeted cellular metagenomes

The phylogenetic placement, functional clustering and experimental design, which excluded cells with chlorophyll fluorescence, make it likely that three of the four viruses are viruses of heterotrophic protists, especially PacV-1605, found in association with the uncultivated choanoflagellate *B. minor*. Multi-cell sorts, depending on the sorting parameters and gating can capture mixed taxa, as is the case here. Hence, these sorts are more restrictive in the information they provide on co-association between specific host cell and virus. For the multi-cell sorts here, while one could conjecture that the more relatively abundant protistan taxa in each might represent the viral host, i.e. a Syndiniales (67-155, oPacV-662 and oPacV-421) and a MAST-4 (Meso1, mPacV-611), it could also be argued that one of the taxa of lesser abundance was infected, with most population members already having being lysed, leading to lower relative abundances. Notably, despite being sorted in a mixed population of heterotrophic protists, the phylogenetic and functional characterization oPacV-421 (based on the overall presence and absence patterns of orthogroups) suggests it is likely to be a virus of a eukaryotic alga. It is possible that the host of oPacV-421 had been previously consumed by a heterotroph present in the multi-cell sort, or otherwise the virus was associated with a senescing photosynthetic cell that had reduced chlorophyll fluorescence. An additional possibility is that perhaps an infected cell was acidified during infection, as observed during Tupanvirus infections [6], possibly resulting in a positive Lysotracker signal.

Culture-independent methods leave some questions open, such as, do the individual PacVs represent single entities? Or closely-related populations assembled into a single genome? The experimental design and lack of underlying sequence diversity, e.g. single nucleotide variations, make it likely that each of the PacV genomes represent individual biological entities (see electronic supplementary material, Discussion). A second question is whether these viral genomes could be remnants of ancient infections that are integrated in eukaryotic hosts. This is unlikely to be the case, based on characteristics of such remnants that have been described elsewhere, such as low coding density and GC-content that is similar to the hosts (see electronic supplementary material, Discussion).

### (b) Evolutionary history and distinctive functions of PacVs in the ocean

Phylogenetic reconstructions based on proteins that are considered core to the NCLDV, specifically the ten proteins used here from the putatively ancestral set and Family B DNA Polymerase, placed cPacV-1605 and mPacV-611 in a statistically supported clade within the *Mimiviridae*, termed here PPVC and comprised of deeply branching members, including CroV and the Faunusvirus. The placement of oPacV-662 remains to be resolved, but it may eventually form an independent lineage within the *Mimiviridae*. oPacV-421 was affiliated with a group of algal *Mimiviridae*. As more genomes become available, we expect resolution and robustness of the overall topology of the *Mimiviridae* tree to improve. However, our analysis definitively resolves a new statistically supported clade, PPVC, which likely represents a family-level division from previously recognized NCLDV groups (figure 2; electronic supplementary material, figure S9). The latter have received a variety of classification levels for which a consensus has yet to arise. Given the deep branching within the PPVC clade, it further seems likely that its members represent multiple NCLDV subfamilies within the PPVC.

With respect to non-'core' NCLDV proteins, the number of sequences with no matches in other genomes, as well as the distribution of affiliations to NCLDV, eukaryotes and prokaryotes observed here has been typical for most NCLDV genomes and metagenomes [5,17,107,108]. The high numbers of novel proteins identified in each virus at least partially the result of the low taxonomic sampling that currently exists for giant viruses. Interestingly, about half of the proteins that do have matches to NCLDV within their top 10 blastp matches also have hits to cellular lineages (figure 3). Thus, with the above caveat on low taxonomic sampling in mind, we postulate that horizontal gene transfer, or host-to-virus gene transfer, and retention, is substantial in the giant viruses.

We identified several proteins that have implications for viral augmentation of metabolic processes that are often thought to limit the growth of both unicellular eukaryotes and prokaryotes in marine environments. One of these was a Rh-like protein that comes from the Amt/Mep/Rh superfamily. This superfamily is made up of three phylogenetically distinct families [87,113] that are involved in ammonium and/or ammonia uptake and/or excretion [110,114]. Across the family, the compound that is transported (e.g., $NH_{4}^{+},$ $NH_{3}^{-3},$ methylammonium) has often not yet been identified. However, based on family members that have been functionally characterized, specificity is high for the target substrate, and not for other monocationic cations, at least in plants and yeast [115]. In contrast to Amt and Mep, findings are less clear for Rh proteins [116], which have also been implicated in CO_2_ transport [117,118]. Rhesus factor proteins have mostly been found in eukaryotes, of which those of mammalian origin have been most studied [110,111]. The ammonium transporters in the viruses sequenced here are closest to those found in choanoflagellates and other heterotrophic marine protists, but fall in a poorly characterized part of the Rh factor clade. Nearly all efforts have focused on mammalian versions and only recently was this region of the tree explored with attention to marine taxa [83]. Thus, the function of the PacV proteins in this family during host infection, as well as the protistan homologs from which they possibly derive, is unknown but likely involved in nitrogen transport, as typical for Amt/MEP/Rh superfamily members.

The two PacVs encoding Rh proteins were recovered from sites where ammonium was detectable and, further, were recovered from depths that had elevated ammonium concentrations (figure 1*e*). This contrasts with the hypothesis that has been made for cyanobacteria and cyanophages suggesting that they retain high-affinity phosphate uptake genes in environments where that nutrient is highly limiting [119–122]. Notably, a member of the Amt superfamily has been reported in another eukaryotic marine virus, OtV6 [83]. This virus infects the prasinophyte alga *Ostreococcus tauri*, which was isolated from a lagoon where high concentrations of nitrogen-related compounds occur [123]. *O. tauri* and other prasinophytes have multiple Amt proteins, with different origins [90]. The version acquired by OtV6 is related to Amt1.1 (XP_022840606.1) of its host, which belongs to a plant and green algal ammonium transporter family [87]. Functional characterization of vAmt showed that expression during infection increased substrate affinity in the host [83]. Similarly, phosphate transporters reported in algal viruses (including *Ostreococcus* viruses) come from environments that are not typically phosphate limited [96]. This implies that retention of these host-acquired proteins is significant in environments where the virus can immediately augment host nutrient acquisition, because the nutrient is available.

cPacV-1605 also encodes a viral rhodopsin (VirR) as well as the biosynthesis pathway for the pigment from which the required chromophore is produced. Together, microbial rhodopsins and their chromophore, retinal, are known to form a light-sensitive photosystem. Different rhodopsin photosystems have distinct functions in cellular organisms, including phototaxis or generation of a proton gradient for energy transfer. The latter can prolong survival of heterotrophic bacteria under starvation conditions if light is available [124,125,126]. The only cultured virus with a known host that harbours a microbial rhodopsin is PgV [42], though viral rhodopsins have been noted in multiple metagenomic studies, e.g. [25,127,128]. The only other viruses that have a known host, and VirR, also encode the biosynthesis pathway for beta-carotene/retinal [25]. These are the Choanoviruses [25], a lineage that is placed within a different region of the tree from cPacV-1605 (figure 2), but that was recovered from the same uncultivated predatory heterotrophic protist as cPacV-1605, the uncultivated choanoflagellate *Bicosta minor*.

The functional capabilities of microbial rhodopsins can be in part predicted by three amino acids at specific positions, known as rhodopsin motifs. While the viral rhodopsin type shared by ChoanoV1 and PgV (VirR_DTS_) has been shown to pump protons when expressed in *E. coli* [25], the cPacV-1605 rhodopsin (VirR_DTV_) has a biochemically uncharacterized motif. With respect to VirR, it will be important to understand the cell biological interaction within host systems to fully characterize how they influence host biology and potential photo-heterotrophy.

The functional clustering of giant viruses based on presence and absence patterns of all orthogroups, taking the viruses with known hosts as ‘guides’, but also including uncultured giant viruses with unknown hosts, showed distinctions from clades derived from phylogenetic analyses. For example, the ChoanoViruses [25] group with giant viruses that infect only marine heterotrophic predatory hosts including those in the PPVC, whereas the ChoanoViruses are placed in a different part of the extended *Mimiviridae* region of the tree by phylogenetics. In addition, the Faunusvirus shifts from being grouped in the PPVC clade identified by phylogenetics, to an orthologue-based cluster of giant viruses from known heterotrophic non-marine hosts, such as *B. saltans* virus and other Klosneuviruses [5,34,38,107]. Hosts for the latter are largely unknown, but they probably infect non-photosynthetic protistan hosts since they have been identified from wastewater, deep-sea sediments and soils. At the broadest level, the viruses of the various photosynthetic lineages, such as chlorophytes and prasinophytes, as well as photosynthetic alveolates and stramenopiles, cluster together, to the exclusion of viruses of heterotrophic protists, even those of stramenopile hosts. This suggests that host lifestyle has a strong influence on the genomic repertoire of giant viruses. Collectively, these findings bring forth strong functional similarities between viruses that infect hosts with similar trophic modes, such that host habitat and lifestyle potentially trump phylogenetic relatedness as a determinant of gene repertoire.

### (c) Distribution of PacVs in the ocean

We recovered thousands of exact or nearly exact reads from metatranscriptome mapping to cPacV-1605, recovering nearly all predicted proteins (85%). In contrast, mPacV-611, oPacV-421 and oPacV-662 were not recovered at high identity likely because metatranscriptomes were not available from the samples or oceanic sites from which they were recovered. We rarely recovered sequence matches with high similarity in Tara Oceans data to PacV viruses. Each of these observations is consistent with the idea that these viruses may be endemic to the region in which they were identified, although they are influenced by suitability of sampling practices for recovering giant virus sequences. The PacVs did, however, recruit broadly at 60–80% amino acid identity. The low recruitment at high similarity, but high recruitment at low similarity highlights the vast unexplored diversity of *Mimiviridae* [129]. Interestingly, for double stranded DNA (dsDNA) phages, additional metagenomic sampling recovers relatively little new diversity based on rarefraction curves of metagenomic samples from tropical and temperate oceans [130–132]. Thus, our results indicating that there is considerable undiscovered diversity of giant viruses contrasts with diversity results for dsDNA phage communities in the ocean, which appear to be well-sampled.

## 5. Conclusion

Taken together, our studies show the value of targeted metagenomics based on cell sorting for recovering undersampled viruses that are important evolutionarily and ecologically. These viruses appear to often be obscured in traditional bulk metagenomic data, or difficult to assemble, from environments with high diversity. Isolation, propagation and genome sequencing of these viruses will facilitate additional discoveries and understanding of the virocell [133]—as well as impacts on the host. However, the PacVs from individual and multi-cell sorts, as well as the newly discovered ChoanoViruses [25], do not appear to have cultured hosts. Indeed predatory heterotrophic taxa can be particularly difficult to culture since it requires initial laboratory conditions that are suitable for both the host and the prey community [33].

The targeted metagenomic assemblies provided herein improve possibilities for assigning metagenomic sequences to giant viruses. For example, the PacVs may help assign metagenomic data from Tara, and time-series studies such as the San Pedro Ocean Time-series, the Bermuda Atlantic Time-series Study or Hawaiian Ocean Time-series [134–137], which might otherwise be assigned to cellular organisms or left as unknowns. Our recovery of a second giant virus lineage that infects a heterotrophic protistan host, and carries not only a microbial rhodopsin, but also the biosynthesis pathway for the required pigment and cleavage enzyme [138], is particularly notable. A recent study reported that microbial rhodopsins rival the amount of solar energy capture performed by pigments for oxygenic photosynthesis in some ocean regions. However, this study did not tease apart what fraction of those rhodopsins might be virally derived [139], indeed it was not yet known that giant viruses encoded the entire rhodopsin photosystem, as neither the first study reporting this discovery [25] nor the present study were as yet available. Collectively, our findings raise important questions about host–virus interactions and the possibility for transient or even longer-term mutualism, depending on the extent to which viruses induce immediate lysis or instead coexist within their host. Much of the existing literature on host–virus interactions is based on laboratory experiments at unrealistic titres, and has been performed on smaller hosts (whether eukaryotic or bacterial) that typically have much larger population sizes, at least seasonally, than some of the protists in which giant viruses have been reported. This affects the host–virus encounter rate dynamics and presumably viral strategy. Certainly, to more fully understand the ecological and evolutionary influence of the uncultivated members of the *Mimiviridae* studied herein, further understanding of how they shape the cell biology of their natural hosts will be essential, alongside high-throughput efforts for cultivation and eukaryotic single-cell sequencing approaches.

## Supplementary Material

Supplementary Figures 1-11

## Supplementary Material

Supplementary Discussion

## Supplementary Material

Table S1

## Supplementary Material

Table S2

## Supplementary Material

Table S3

## Supplementary Material

Table S4

## Supplementary Material

Table S5

## Data accessibility

GenBank genome accessions: oPacV-421 (MN448291-MN448303), oPacV-662 (MN448266-MN448266), mPacV-611 (MN448284-MN448290), cPacV-1605 (CABVLZ010000001-CABVLZ010000014).
