# Supplementary material for: Targeted metagenomic recovery of four divergent viruses reveals shared and distinctive characteristics of giant viruses of marine eukaryotes
Source: Philos Trans R Soc Lond B Biol Sci. 2019 Oct 7;374(1786):20190086. doi: 10.1098/rstb.2019.0086 (PMC6792449; doi:10.1098/rstb.2019.0086)
Supplement: Supplementary Figures 1-11 [file rstb20190086supp1.pdf]

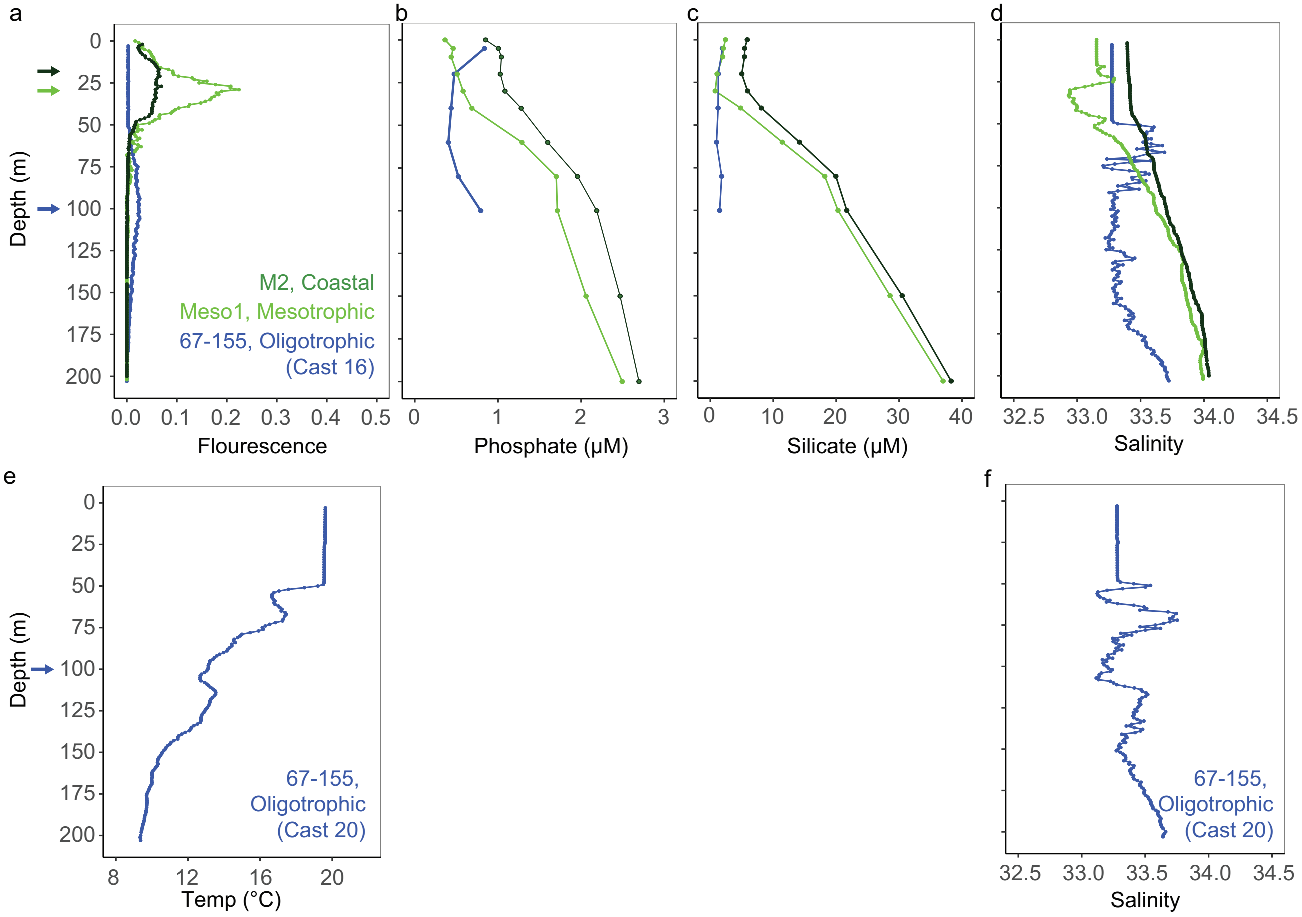

Supplementary Figure 1: Depth profiles of environmental parameters for sampling locations from which the PacV sequences were obtained: (a) CTD fluorescence, (b) phosphate, (c) silicate, and (d) salinity. As in Figure 1b-e, the arrows indicate the depth from which water was collected for flow cytometric sorting, which was from the deep chlorophyll maximum for 67-155 and at the sub-surface maxima for the two other sites. Note values for CTD fluorescence are not directly comparable between locations due to calibration inconsistencies. However, within a cast, the values are informative. Thus, the profile at 67-155 demonstrates that the sample was taken from the deep chlorophyll maximum depth, complementing the more quantitative chlorophyll measurements, from which the deepest sample came from 100 m (Figure 1c). Note, profiles for station 67-155 used in the main text are generally (Figure 1b-d) from a CTD cast 8.5 hours before the CTD cast from which the cell sorting was performed except for the ammonium data (Figure 1e), which is from 25.5 hours prior to cell sorting. These casts were displayed in the main text because nutrients were collected from these earlier CTD casts. For comparison, we show the profiles for (f) temperature and (g) salinity from which the sorts were performed ("Cast 20"), here, which shows the temperature and salinity are highly similar to the earlier casts (shown in Figure 1b and (d), here).

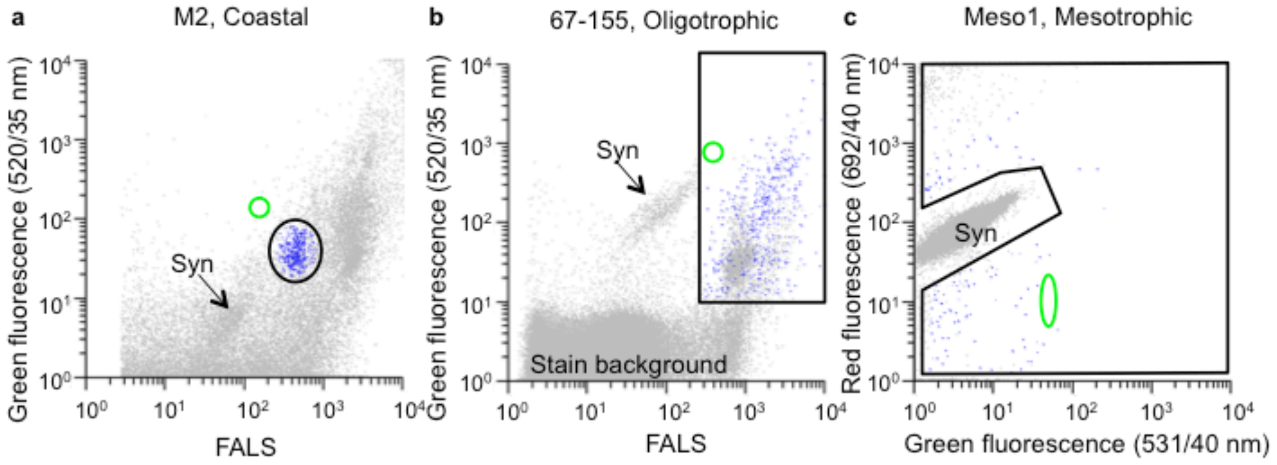

Supplementary Figure 2: Fluorescence activated cell-sorting strategy of samples where NCLDVs were recovered. Sorted cells (blue dots) were selected based on positive LysoTracker green signal captured by a 520/35 nm or a 531/40 nm bandpass filter, with variable stringency (black windows), for stations a.) M2, b.) 67-155, and c.) Meso1. *Synechococcus* cells and most of the photosynthetic eukaryotes were identified and excluded by their orange and red fluorescence from phycoerythrin and chlorophyll pigments (captured by 572/27 nm and 692/40 nm bandpass filters), respectively, and using the Forward Angle Light Scatter (FALS) parameter as a proxy for cell size (windows not shown). Green circles represent the position of 0.75 µm yellow-green beads. Note, in order to prevent addition of extraneous DNA or contamination, yellow-green beads (i.e., internal standards) were not added to samples used for cell sorting; their position was determined on preparative flow cytometry runs prior to cell sorting.

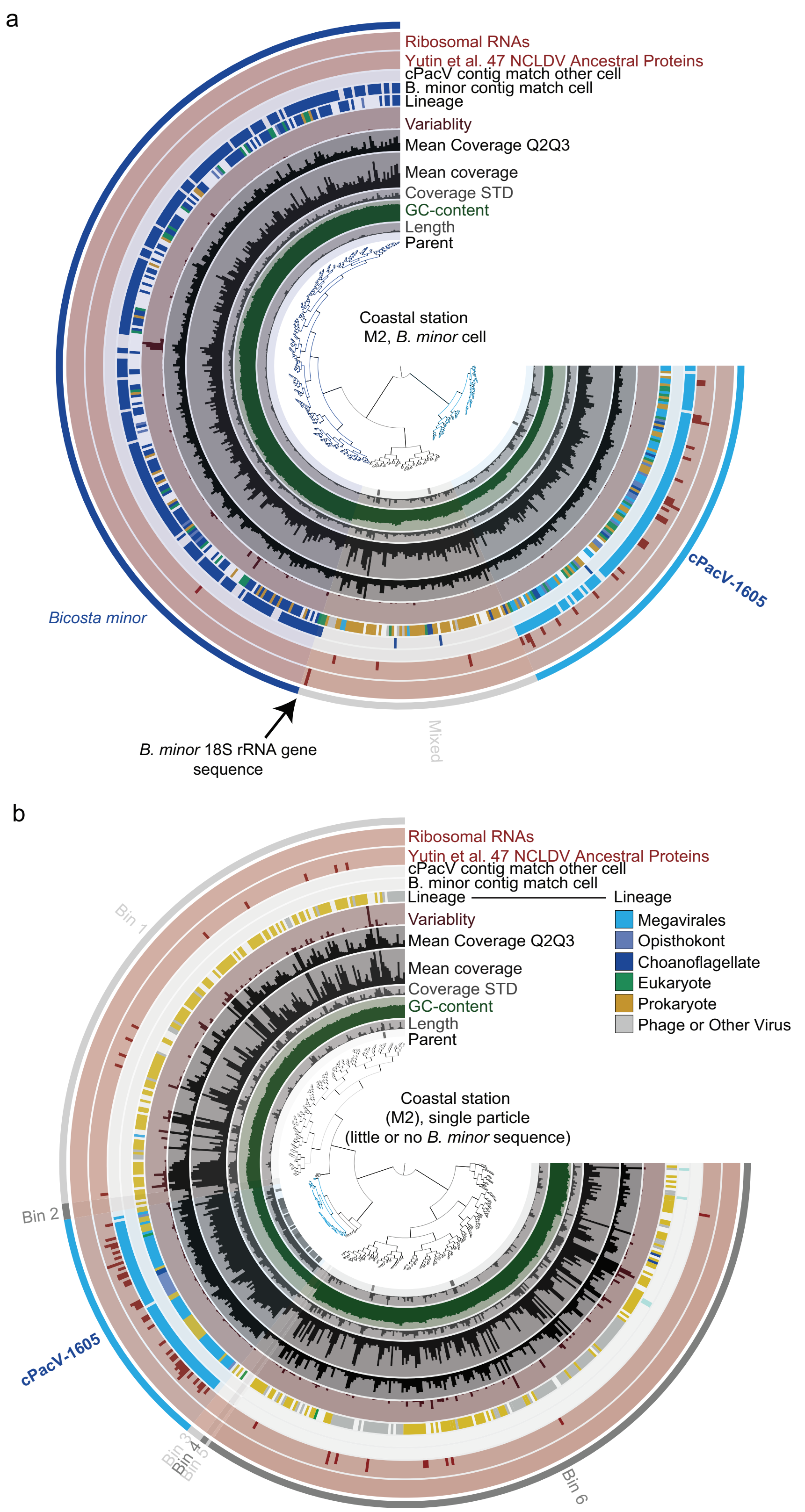

Supplementary Figure 3: cPacV-1605 recovery and comparison between two singlparticle sorts. (a) and (b) Anvi'o diagrams showing the clustering of contigs by tetranucleotide

frequency for manual binning for the sort with (a) a full *Bicosta minor* 18S rRNA gene sequence (b) no recovered 18S rRNA and. The outermost layer shows which “Bin” the contigs were sorted. The second and third layer shows the contigs which contain rRNA gene sequences, and the number of the predicted proteins with matches to the 47 putatively ancestral NCLDV proteins [50], respectively. The fourth layer shows the contigs (the contigs marked with a blue section) that had a match in the other sample. That is, the blue sections in layer four indicate cPacV-1605 contigs in (a) that had a blastn match of >250 bp and >99% nucleotide ID to a contig from the cPacV-1605 bin from (b), and vice versa). Similarly, the fifth layer shows contigs that had > 95% nucleotide similarity to a four-well B. minor draft genome (686 in (a), but only four such contigs in (b)). Layer 6 indicates the lineage of the best blastx hit in NCBI nr or a recently available database of choanoflagellate transcriptomes (corresponding legend at bottom left). Layer 7 indicates the “variability” or single nucleotide variation of the contigs, with a maximum of 1.7 bp/Kb and 5.4 bp/Kb in (a) and (b), respectively. Maximum values of variability for cPacV1605 were 0.5 bp/Kb and 1.6 bp/Kb and for (a) and (b), respectively. Layer 8 and 9 rows show the mean coverage of the second and third quartiles of each contig, and the overall mean coverage, respectively. Layer 10 shows the standard deviation of the coverage along each contig. Layer 11 shows the GC-content of each contig. Layer 12 shows the length of each contig. Layer 13, for binning, contigs were split into 20 Kb “splits” so that contigs longer than 20 Kb can be more fairly compared to the other contigs. Thus, layer 13 shows, if a contig was split due to a length of greater than 20 Kb which of the contigs belong to the same original contig. The “Parent” row indicates which splits were a part of larger contigs, where the same gray shading intensity of adjacent contigs indicates they are from the same contig. The contigs (all > 1 Kb) are clustered by tetranucleotide frequency according the dendrogram (center of circle).

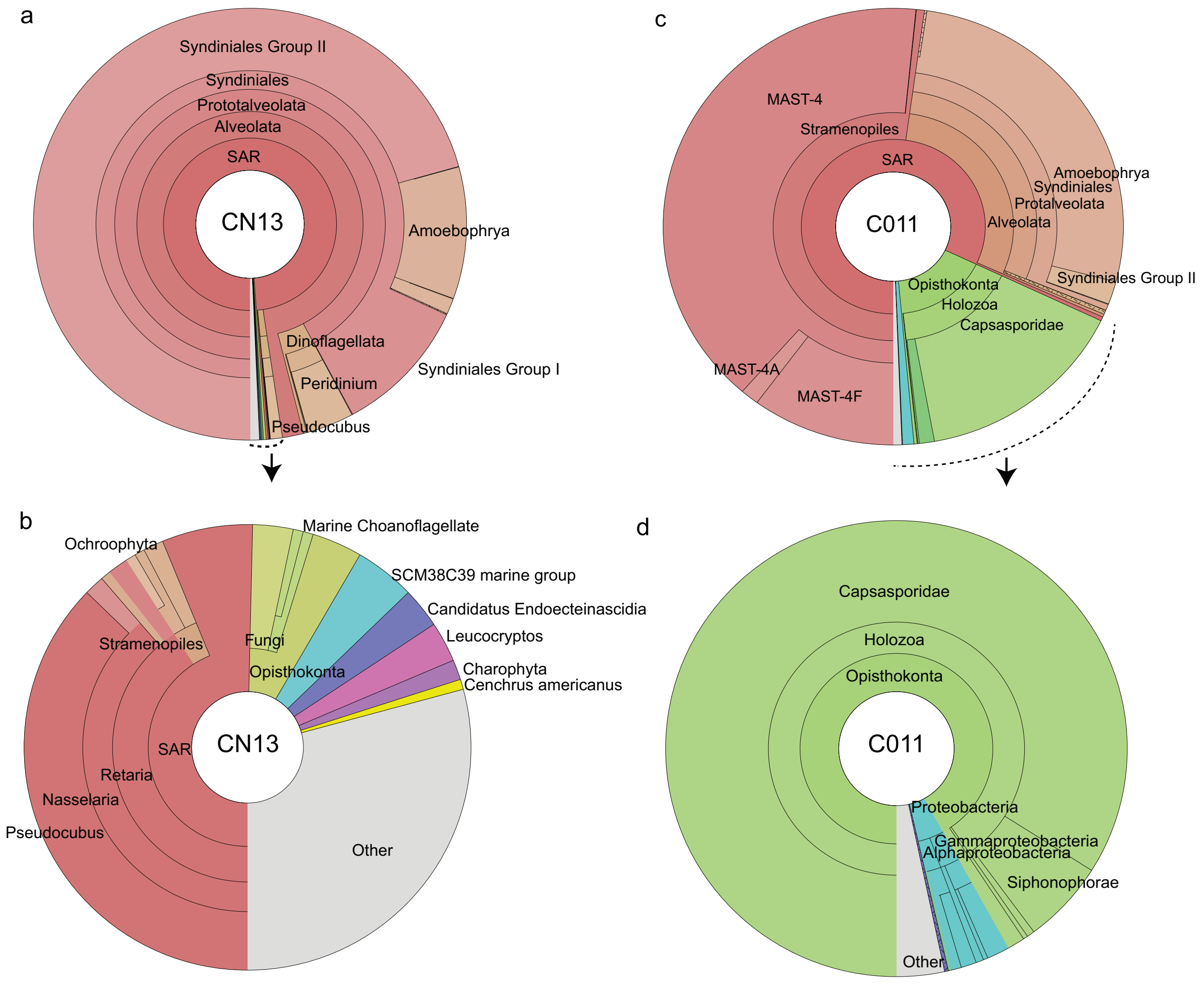

Supplementary Figure 4: Classifications and proportions of rRNA gene in multi-cell sorts. The proportions of the Krona indicate the number of rRNA gene sequences with that classification for (a) all rRNA gene sequence reads from 67-155, (b) minor components from 67-155, (c) all rRNA reads from Meso1 and (d) minor components from Meso1.

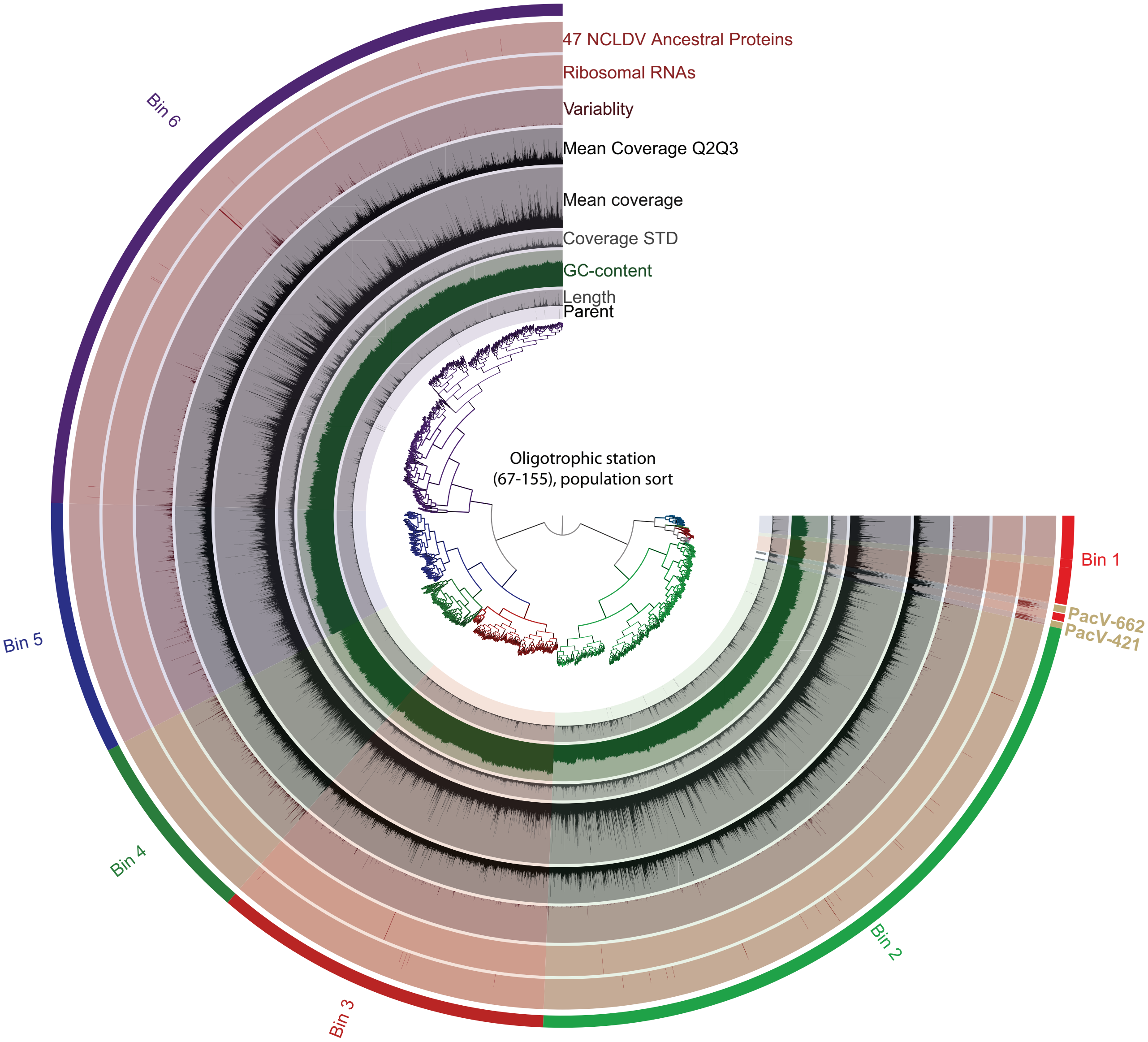

Supplementary Figure 5: Visualization of the contigs assembled from the multi-cell sort from which in which oPacV-662 and oPacV-421 were recovered. All layers are as described in Supplementary Figure 3, but excluding layers four, five, and six from figure Supplementary Figure 3. For “zoomed-in” view of Bin 1 (in red) and oPacV-662 and oPacV-421 contigs, see Supplementary Figure 6.

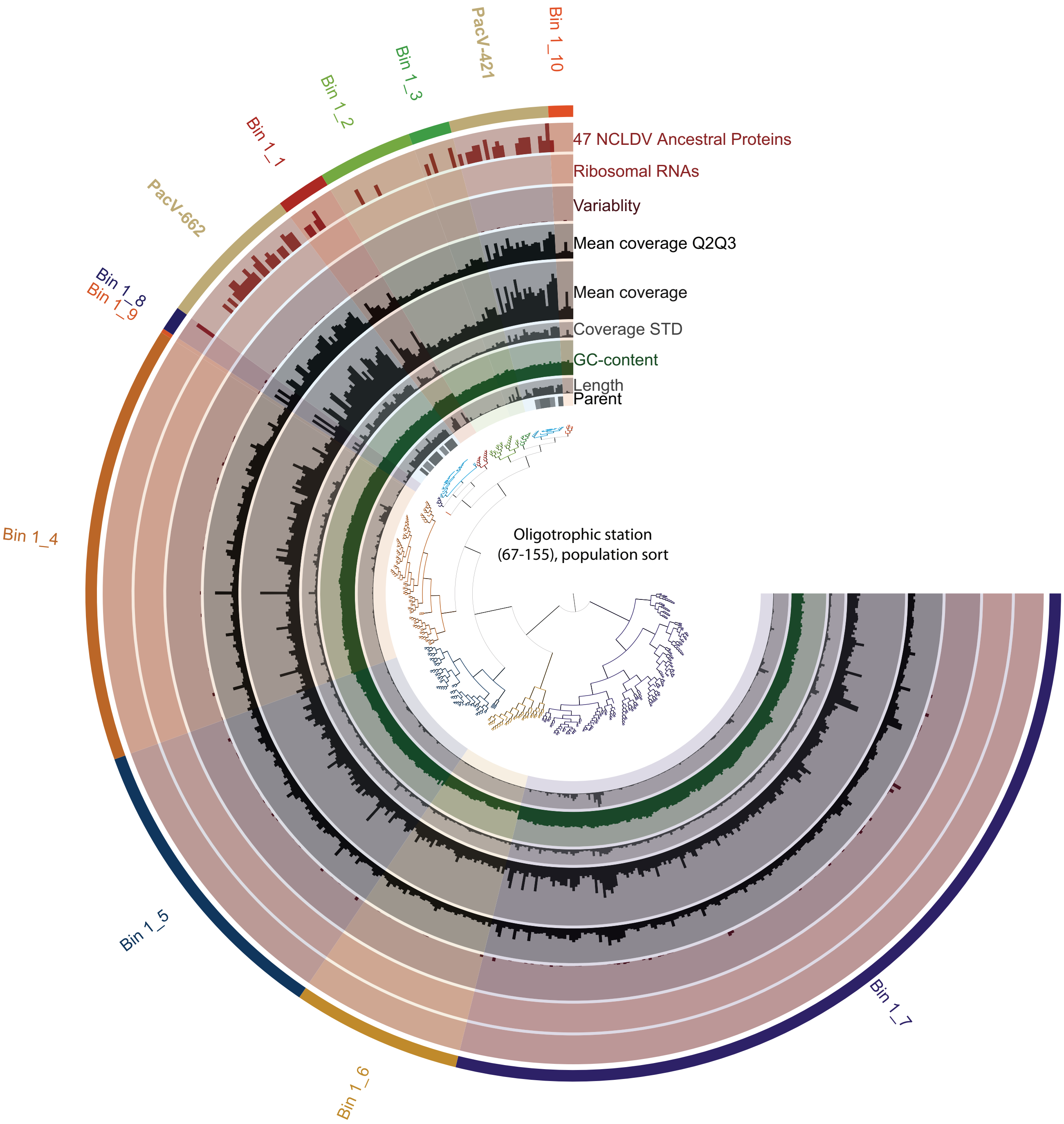

Supplementary Figure 6: Visualization of the "zoomed-in" view of Bin 1 and oPacV-662 and oPacV-421 contigs with all layers as described in Supplementary Figure 3, excluding layers four, five, and six.

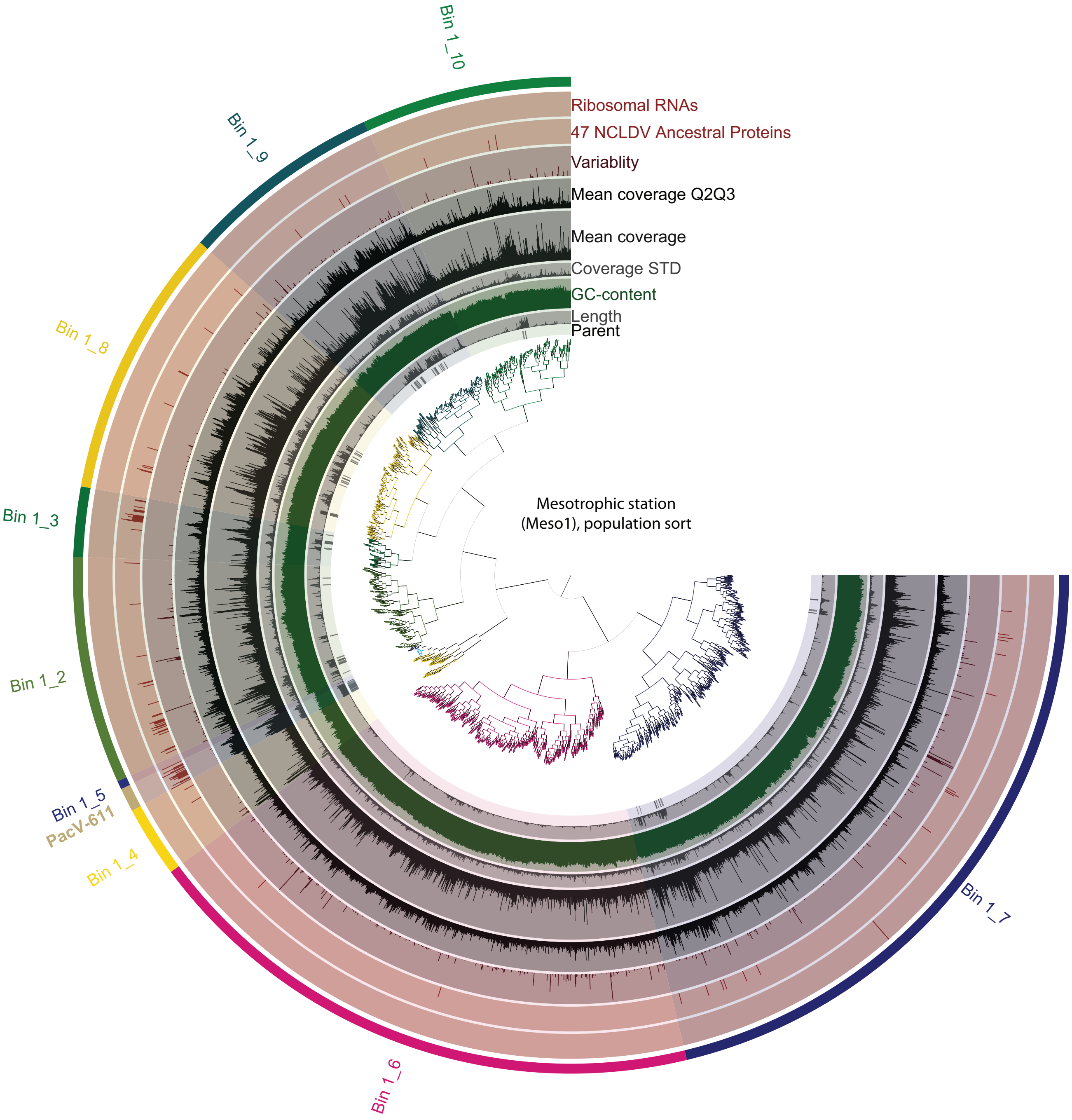

Supplementary Figure 7: Visualization of a "zoomed-in" view of a group of contigs with relatively low GC-content from a multi-cell sort from in which mPacV-611 was recovered. Layers are all as described in Supplementary Figure 3, excluding layers four, five, and six.

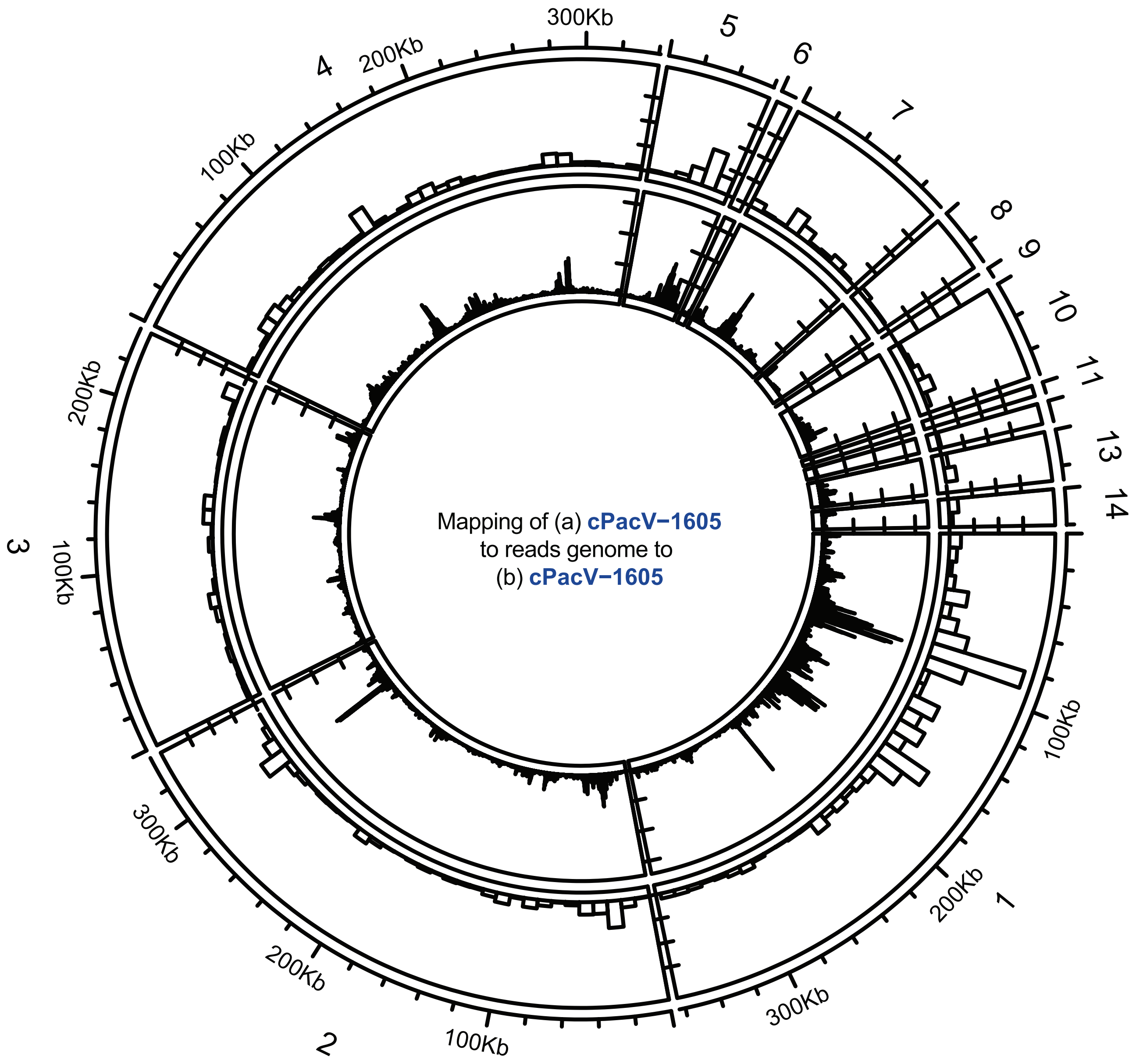

Supplementary Figure 8: High stringency mapping (> 99% similarity) of the *B. minor* single cell (Supplementary Figure 3a) to the cPacV-1605 genome that we used for downstream analyses (Figure 4a, Supplementary Figure 3b). The figure shows mapping coverage at bin increments of 10 Kb and 150 bp for the outer and inner layers, respectively. Average coverage was 40.5x.

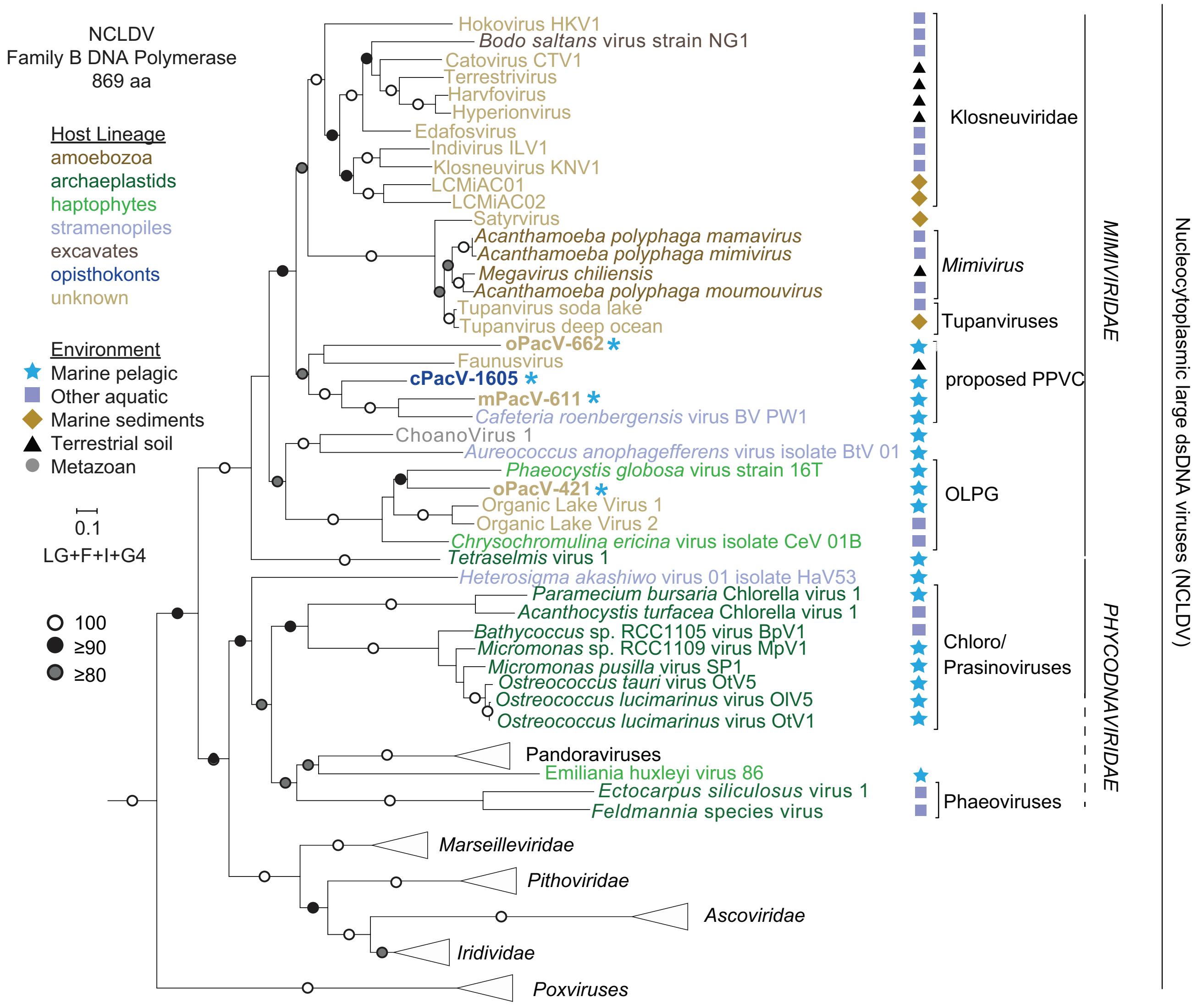

Supplementary Figure 9: Maximum likelihood phylogenetic reconstruction of genome sequenced NCLDV based on the Family B DNA Polymerase protein. Maximum likelihood methods were used to perform reconstructions on an 869 amino acid alignment that included the four novel NCLDV described here (indicated by asterisks) and representative sequences from genome or metagenome sequenced NCLDV. Faustoviruses are excluded [137]. Bootstrap support reflects the percentage from 1000 ultrafast bootstraps. The text color of each virus is by host lineage if known, and the type of environment from which each virus was recovered is shown by colored symbols to the right.

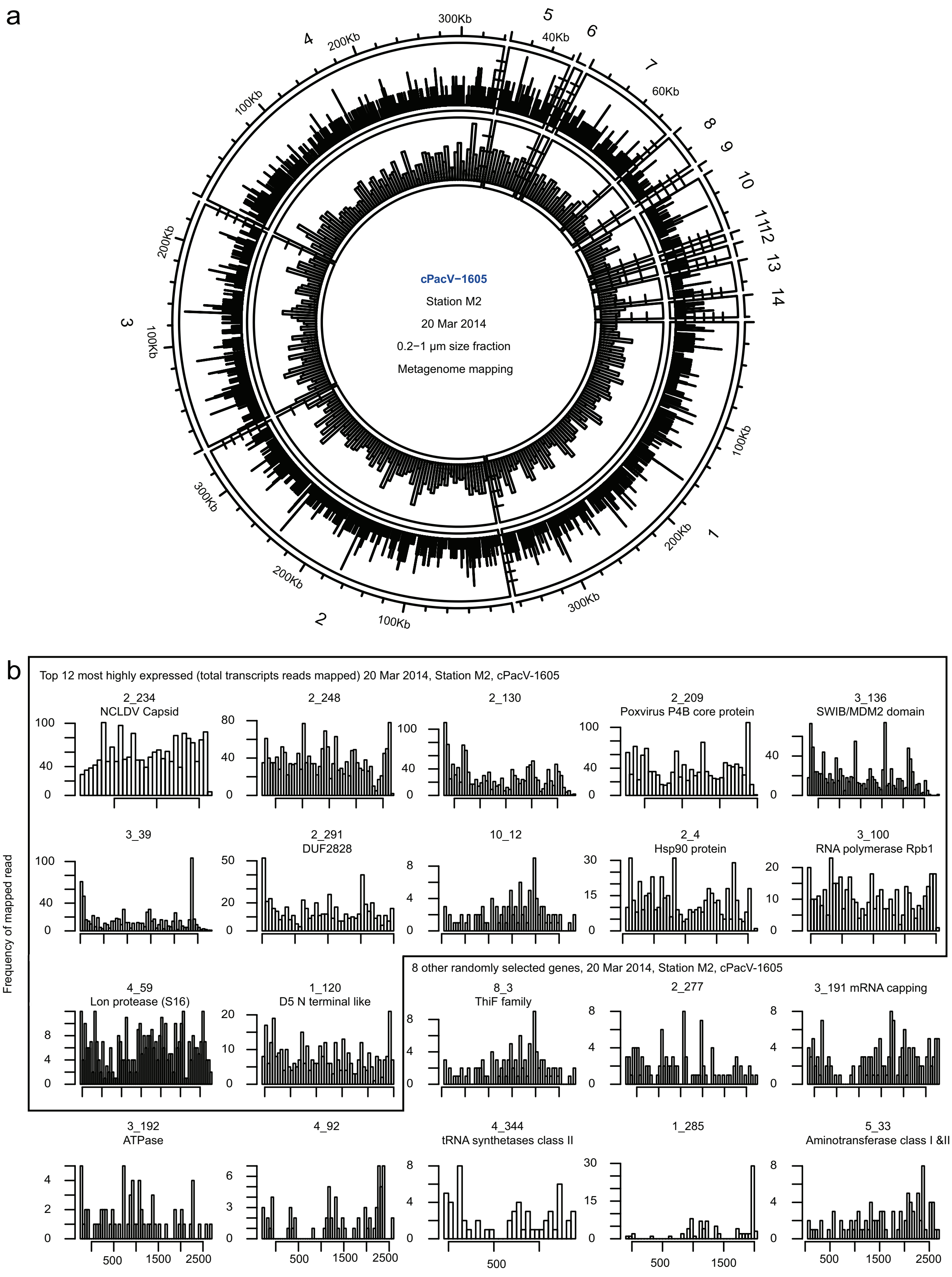

Supplementary Figure 10: Mapped reads (>99%) to cPacV-1605 are highly uniform across both genes (via transcriptome) and the genome (via a metagenome). (a) A

metagenome collected at the same station (M2) and date of the sorting experiment from which cPacV-1605 was recovered shows low, but even, coverage across the full cPacV-1605 genome. Reads were mapped with bbmap at 99% sequence similarity. For the outer and inner layers the reads were placed on the genome map by the starting point of the mapped read at 150 bp and 10,000 bp increments, respectively. (b) The coverage of the 12 most highly expressed genes, and a random selection of 8 other genes indicates generally even coverage across the genes. This suggests the mapping is specific and not an artifact of mapping to highly conserved regions. Tick marks along the x-axis represent 500 bp. For each gene, reads were binned into 50 separate bins, resulting in the histograms shown.

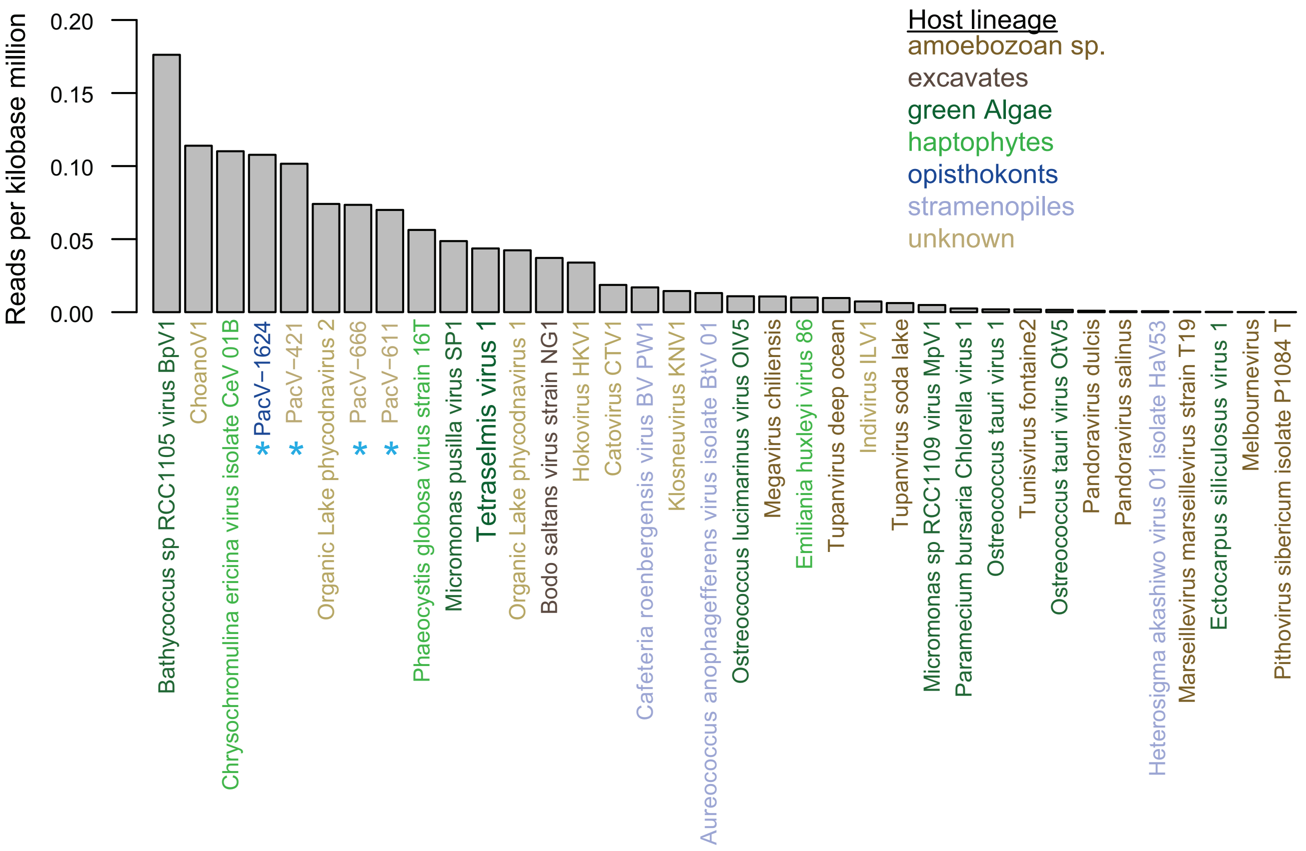

Supplementary Figure 11: Metatranscriptomic read recruitment from Tara Oceans. Forward and reverse reads were mapped from 87 Tara Oceans sites. Reads were initially recruited to a set of 36 NCLDV genomes with diamond blastx with a bit-score cutoff of 50. Reads with bit-score over 50 were subsequently also searched against NCBI nr. Then, all the matches (both NCLDV and NCBI) were compared and the best hits (by bit-score) were selected. In the event of ties, the best hit was chosen randomly.
