# Supplementary material for: Targeted metagenomic recovery of four divergent viruses reveals shared and distinctive characteristics of giant viruses of marine eukaryotes
Source: Philos Trans R Soc Lond B Biol Sci. 2019 Oct 7;374(1786):20190086. doi: 10.1098/rstb.2019.0086 (PMC6792449; doi:10.1098/rstb.2019.0086)
Supplement: Supplementary Discussion [file rstb20190086supp2.docx]

**Targeted metagenomic recovery of four divergent viruses reveals shared and distinctive characteristics of giant viruses of marine eukaryotes**

David M. Needham^1^, Camille Poirier^1,2^, Elisabeth Hehenberger^1,2^, Valeria Jiménez^1,+^, Jarred E. Swalwell^3^, Alyson E. Santoro^4^, Alexandra Z. Worden^1,2^

^1^Monterey Bay Aquarium Research Institute, Moss Landing, CA, USA;

^2^Ocean EcoSystems Biology Unit, RD3, GEOMAR Helmholtz Centre for Ocean Research, Kiel, Germany;

^3^School of Oceanography, University of Washington, Box 357940, Seattle, WA, USA

^4^Department of Ecology, Evolution and Marine Biology, University of California Santa Barbara, CA, USA

^+^, now at : Sorbonne Université, CNRS, Station Biologique de Roscoff, Roscoff, France;

**Supplementary Discussion:**

Notably, 3 (of 13 total contigs) from oPacV-421 and 4 (of 18 total contigs) from oPacV-662 were shorter than 5 Kb. These contigs make up 3% and 1.3% of the partial genomes, respectively. Their short length may decrease the confidence of the inclusion in the genome bins, in particular, since coverage information should not contribute to binning due to bias of MDA amplification. However, given the nature of the reduced complexity of targeted-metagenomes (relative to bulk metagenomes), very low GC-content, and tetranucleotide frequency clustering we chose to include them in our genomic bins (Figure 4, Supplementary Figure 4 and 5). None of the major features that we describe are located on these smaller contigs (Figure 4). Thus, their contribution is relatively minimal, yet ultimately they lend to more complete genomic information.

A variety of evidence lends support to the idea that the collections of contigs are from a single entity: 1.) their highly tetranucleotide frequencies and GC-content that is highly distinct from co-sorted cellular DNA and shared within a given PacV, 2.) single copy of the only single copy marker, PolB, and 3.) a lack of underlying sequence diversity within the genome bins (no greater than expected from MDA or sequence error). Additionally, in the case of PacV-1605, which we recovered twice, 91.2% of the larger version of the genome was recovered in the other well, indicating the high likelihood the contigs come from a single virus population. Missing fragments are likely due to differences in coverage of the genomes (621x vs 33x), bias in MDA amplification. The very low sequence differences (<1% nucleotide dissimilarity of aligned regions) are consistent with potential errors from sequencing or assembly.

For the multi-cell sorts, and to a lesser degree single cell sorts, one potential complication of the binning approach is that multiple giant viruses may have similar GC-content and, as a consequence, convergent tetra-nucleotide frequencies challenging the ability to distinguish different viruses. Additionally, for all viruses, our examination of the copy numbers of the 47 ancestral Megavirales viruses, i.e., proteins that are commonly found in single copy with Megavirales, suggested the PacV each came from a single virus or population. Specifically, all 19 of these proteins that are only ever found in single copy in complete giant Megavirales genomes are also only found once in PacV genome assemblies (Supplementary Table 2). Further, that the ancestral viral proteins were relatively evenly distributed across each of the viral contigs assigned to PacVs and the underlying very low level of single nucleotide variation (in line with sequencing error rates) suggests the partial genomes are from a single virus population and/or a single virus population from an infected cell.

Detection of giant virus-derived genes in eukaryotic genomes is rather common, especially in aquatic protists; i.e., integrated genomic fragments from ancient infections (1, 2). The genomic regions surrounding such viral genes are typically small (a few genes up to tens of Kb (3–5)), but have been reported to reach up to ~400 Kb (1). However, the few large contigs (> 100 Kb) that have been reported tend to be characterized by low coding density (1, 2), transcriptional silence (3, 5), and GC-content similar to the host (due to their long-coexistence) (1, 6). In contrast, the PacV partial genomes are typically composed of contigs > 100 kb (Table 1), are gene dense (~1 gene per kb), have GC-content that is much lower than co-sorted potential hosts. Finally, in the case of PacV-1605 we detected transcription for 85% of the genes at the time of sampling, and absence of the genome in some *B. minor* cells, suggests it is not an integrated remnant in *B. minor* itself. Based on these observations, it likely that the PacV genomes come from contemporary viral infectious units, and not ancient remnants integrated into eukaryotic genomes.

Supplementary References

1. Filée J (2014) Multiple occurrences of giant virus core genes acquired by eukaryotic genomes: The visible part of the iceberg? *Virology* 466–467:53–59.

2. Gallot-Lavallée L, Blanc G (2017) A Glimpse of Nucleo-Cytoplasmic Large DNA Virus Biodiversity through the Eukaryotic Genomics Window. *Viruses*  9(1).

3. Blanc G, Gallot-Lavallée L, Maumus F (2015) Provirophages in the Bigelowiella genome bear testimony to past encounters with giant viruses. *Proc Natl Acad Sci U S A* 112(38):E5318–E5326.

4. Maumus F, Epert A, Nogué F, Blanc G (2014) Plant genomes enclose footprints of past infections by giant virus relatives. *Nat Commun* 5:4268.

5. Maumus F, Blanc G (2016) Study of Gene Trafficking between Acanthamoeba and Giant Viruses Suggests an Undiscovered Family of Amoeba-Infecting Viruses. *Genome Biol Evol* 8(11):3351–3363.

6. Delaroque N, Boland W (2008) The genome of the brown alga Ectocarpus siliculosuscontains a series of viral DNA pieces, suggesting an ancient association with large dsDNA viruses. *BMC Evol Biol* 8(1):110.
